# Klf4 Is a Transcriptional Regulator of Genes Critical for EMT, Including Jnk1 (*Mapk8*)

**DOI:** 10.1371/journal.pone.0057329

**Published:** 2013-02-25

**Authors:** Neha Tiwari, Nathalie Meyer-Schaller, Phil Arnold, Helena Antoniadis, Mikhail Pachkov, Erik van Nimwegen, Gerhard Christofori

**Affiliations:** 1 Department of Biomedicine, University of Basel, Basel, Switzerland; 2 Biozentrum, University of Basel, and Swiss Institute of Bioinformatics, Basel, Switzerland; AMS Biotechnology, United Kingdom

## Abstract

We have identified the zinc-finger transcription factor Kruppel-like factor 4 (Klf4) among the transcription factors that are significantly downregulated in their expression during epithelial-mesenchymal transition (EMT) in mammary epithelial cells and in breast cancer cells. Loss and gain of function experiments demonstrate that the down-regulation of Klf4 expression is required for the induction of EMT *in vitro* and for metastasis *in vivo*. In addition, reduced Klf4 expression correlates with shorter disease-free survival of subsets of breast cancer patients. Yet, reduced expression of Klf4 also induces apoptosis in cells undergoing TGFβ-induced EMT. Chromatin immunoprecipitation/deep-sequencing in combination with gene expression profiling reveals direct Klf4 target genes, including E-cadherin (*Cdh1*), N-cadherin (*Cdh2*), vimentin (*Vim*), β-catenin (*Ctnnb1*), VEGF-A (*Vegfa*), endothelin-1 (*Edn1*) and Jnk1 (*Mapk8*). Thereby, Klf4 acts as a transcriptional activator of epithelial genes and as a repressor of mesenchymal genes. Specifically, increased expression of Jnk1 (*Mapk8*) upon down-regulation of its transcriptional repressor Klf4 is required for EMT cell migration and for the induction of apoptosis. The data demonstrate a central role of Klf4 in the maintenance of epithelial cell differentiation and the prevention of EMT and metastasis.

## Introduction

Epithelial to mesenchymal transition (EMT) involves the loss of epithelial-cell markers and gain of mesenchymal-cell markers at the invasive front of various solid tumors and constitutes a central step during carcinogenesis [1], [2], [3], [4], [5]. Acquiring mesenchymal properties liberates these cells from the primary tumor and allows them to invade neighboring tissue, to enter the circulation and to colonize distant organs. The dramatic changes in cell morphology and behavior during the process of EMT and metastasis are accompanied by substantial changes in gene expression. Recently, a number of transcription factors have been identified that play critical roles in the initiation and execution of EMT and in the metastatic process, including Snail1 (Snail), Snail2 (Slug), Zeb1 (δEF1), Zeb2 (Sip1), E47, Twist, goosecoid, FoxC2, Dlx2, RBPjκ, Yap/Taz, Sox9 and NFκB [6], [7], [8]. However, the activities of these transcriptional regulators do not explain the full range of changes in gene expression during the multistage process of EMT, indicating that additional transcription factors with master control functions must exist.

We have previously established a list of genes that change in their expression during the consecutive morphological states of TGFβ-induced EMT in normal mammary epithelial cells (NMuMG) [9]. This analysis identified Kruppel-like factor 4 (Klf4) as a transcription factor that is reduced in its expression during TGFβ-induced EMT. Klf4 is a zinc-finger transcription factor which is usually expressed in growth-arrested cells and in differentiated cells of the colon, small intestine, testis and lung [10]. Notably, the expression of Klf4 is down-regulated in several different cancer types [11], [12], [13], [14], [15], [16], [17]. Yet, Klf4 has also been found highly expressed in dysplastic epithelium, in breast cancers and in squamous cell carcinoma of the oropharynx [18]. Moreover, Klf4 has been shown to act as an oncogene cooperating with c-Myc in the transformation of cells, and the inducible expression of Klf4 in mice is sufficient to provoke skin dysplasia and squamous cell carcinoma [19], [20]. In contrast, database-mining analysis reveals a correlation between low Klf4 expression and an increased incidence of malignant breast carcinoma [21]. Accordingly, Klf4 exhibits a dual function as a transcriptional activator as well as a repressor at the promoter of various genes in a context-dependent manner [22]. For example, Klf4 executes its oncogenic activity by directly binding to and repressing the p53 gene promoter. In contrast, it acts as a tumor suppressor by binding to and activating the promoter of the p21 cell cycle inhibitor gene [22]. Interestingly, together with Oct4, Sox2 and c-Myc, Klf4 is a pivotal factor in the generation of induced pluripotent cells [23] and is required for the epigenetic reprogramming of a somatic genome [24]. Klf4 knockout mice die shortly after birth due to the loss of skin barrier function as a result of the defective differentiation of the cornified envelope [25]. Similarly, Klf4 is required to maintain the cell morphology of mammary epithelial cells: while its loss induces EMT-like morphological changes, forced expression of Klf4 in invasive breast cancer cells induces epithelial differentiation by directly repressing the expression of Snail1, a potent repressor of E-cadherin gene expression, and by directly binding to the E-cadherin promoter and up-regulating E-cadherin expression [26]. However, other direct Klf4 target genes and the molecular mechanisms underlying Klf4’s functional contribution to EMT and metastasis have remained elusive.

Here, we show that Klf4 is essential for the maintenance of epithelial differentiation and that the loss of its function is required and in part sufficient to induce EMT and metastasis. A combinatorial approach of gene expression profiling and chromatin immunoprecipitation/deep sequencing (ChIP-Seq) analysis revealed many key EMT genes as direct transcriptional targets of Klf4, including N-cadherin (*Cdh2*), vimentin (*Vim*), β-catenin (*Ctnnb1*), VEGF-A (*Vegfa*), endothelin-1 (*Edn1*) and Jnk1 (*Mapk8*). Of these, Jnk1 plays a central role in mediating Klf4-controlled EMT, migration and apoptosis.

## Results

### Identification of Klf4 as a Transcriptional Regulator of EMT

**Figure 1:**
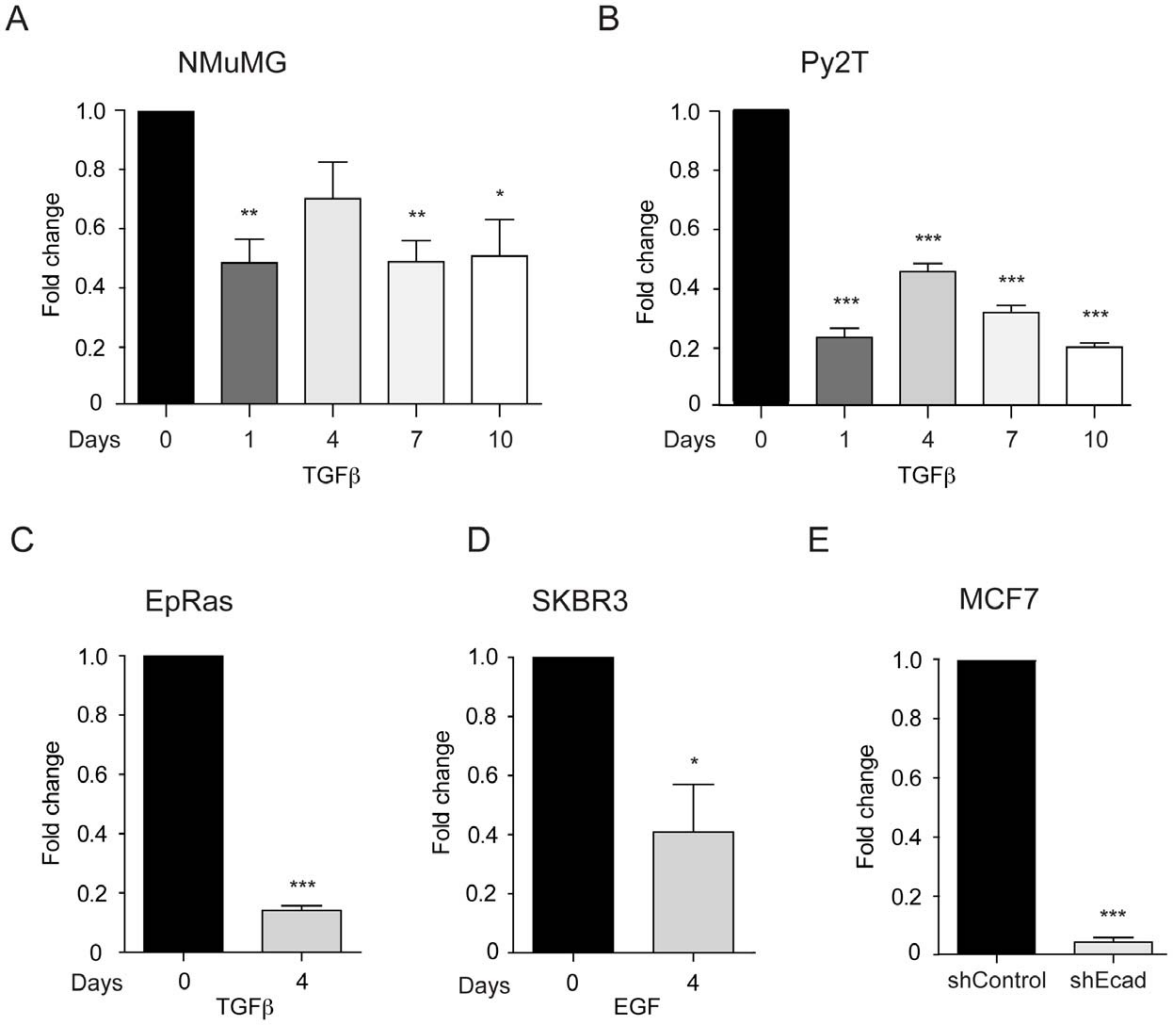
Klf4 expression is down-regulated during EMT. (**A–C**) Klf4 mRNA levels were quantified by quantitative RT-PCR during TGFβ-induced EMT in NMuMG cells (A), Py2T cells (B) and EpRas cells (C). (**D**) Klf4 levels were quantified by quantitative RT-PCR in SKBR3 cells after EGF-induced EMT. SKBR3 cells were treated with 25 ng/ml EGF for 4 days. Shown are the fold-changes as compared to day 0 (no EGF). (**E**) Quantitative RT-PCR analysis of Klf4 mRNA levels in MCF7 cells either stably expressing a control shRNA (shControl) or an shRNA against E-cadherin (shEcad). Statistical values were calculated using a paired, two-tailed t-test and experiments were performed at least three times. * = p≤0.05; ** = p≤0.01; *** = p≤0.001.

We have utilized an established model of EMT, the untransformed normal murine mammary gland cell line NMuMG, to decipher the gene regulatory mechanisms underlying EMT [9]. NMuMG cells undergo progressive EMT upon TGFβ treatment and acquire a complete mesenchymal morphology by more than 10 days of TGFβ treatment (Figure S1A). Genome-wide gene expression analysis of NMuMG cells during TGFβ-induced EMT identified differentially expressed genes at the respective stages. Amongst a large number of genes changing in their expression during EMT, Klf4 mRNA and protein levels were found down-regulated already at day 1 of TGFβ treatment (Figure 1A; Figure S1B). Decreased expression of Klf4 during TGFβ-induced EMT was also confirmed in the murine breast cancer cell line Py2T, which has been established from a breast tumor of MMTV-PyMT transgenic mice [27], as well as in EpRas cells (Figure 1B–C, Figure S1B). Similar to NMuMG cells, both of these cell types undergo EMT upon TGFβ treatment. Klf4 expression was also found reduced in human breast cancer cell lines, such as in SKBR3 upon EGF-induced EMT and in MCF7 cells which undergo EMT upon stable depletion of E-cadherin (Figure 1D, E) [9]. Notably, the levels of Klf4 mRNA were not reduced either upon TGFβ-treatment in NMuMG cells stably depleted for Smad4 (shSmad4 [28]; Figure S1C) or upon treatment with TGFβR inhibitor in NMuMG and Py2T cells in the absence and presence of TGFβ (Figure S1D, E), suggesting that canonical, Smad4-dependent TGFβ signaling represses Klf4 expression, as observed during TGFβ-induced EMT in NMuMG cells.

### Klf4 Maintains the Epithelial Differentiation Status

**Figure 2:**
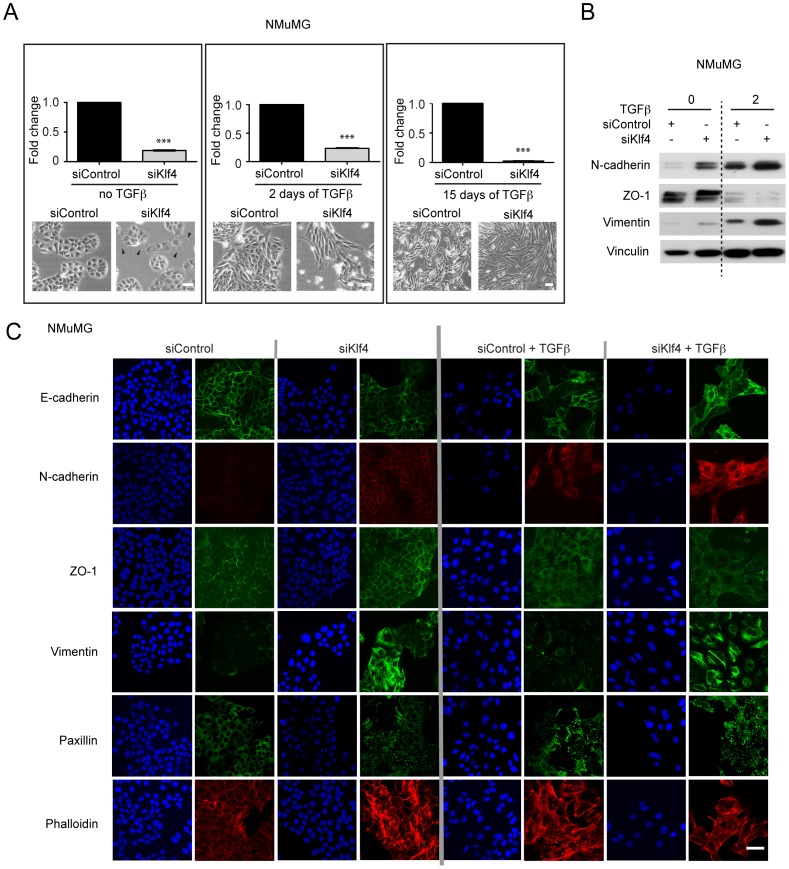
Klf4 maintains epithelial morphology and prevents EMT. (**A**) NMuMG cells were transfected with either control siRNA (siControl) or Klf4-specific siRNA (siKlf4) in the absence as well as in the presence of TGFβ for 2 or 15 days, as indicated. The levels of Klf4 mRNA were determined by quantitative RT-PCR and the morphology of the cells evaluated by phase contrast microscopy. Filopodia and membrane protrusions are marked with closed arrowheads. Size bar, 100 µm. (**B**) Immunoblotting analysis of the expression of the epithelial marker ZO-1 and the mesenchymal markers N-cadherin and Vimentin during TGFβ-induced EMT in control NMuMG cells (siControl) and Klf4 depleted cells (siKlf4). (**C**) Immunofluorescence microscopy analysis of changes in the localization and expression levels of EMT markers during EMT. NMuMG cells transfected with either control siRNA (siControl) or with siRNA against Klf4 (siKlf4) were left untreated or treated with TGFβ for 2 days and stained with antibodies against the epithelial markers E-cadherin and ZO-1, against the mesenchymal markers N-cadherin and vimentin, against paxillin to detect focal adhesion plaques, and with phalloidin to visualize the actin cytoskeleton. Size bar, 50 µm. Statistical values were calculated by using a paired, two-tailed t-test and experiments were performed at least three times. *** = p≤0.001.

We next directly assessed the functional contribution of Klf4 to EMT. Transfection of a pool of two siRNAs against Klf4 (siKlf4) efficiently ablated Klf4 expression in NMuMG cells (Figure S2A). In the absence of TGFβ, Klf4 ablation induced modest changes in cell morphology with a slight increase in cell size accompanied with cell flattening, a partial loss of cell-cell adhesion and an increase in filopodia formation (Figure 2A, left panel). Immunoblotting and immunofluorescence microscopy analysis revealed an increase in the expression of mesenchymal markers, such as N-cadherin and vimentin, in siKlf4 cells as compared to control siRNA-transfected cells (siControl; Figure 2B, C, left panels). In addition, focal adhesion and actin stress fiber formation were substantially increased, as visualized by staining with paxillin antibody and fluorescently labeled phalloidin, respectively (Figure 2C). Conversely, the epithelial adherens junction protein E-cadherin was moderately reduced and displaced from the membrane junctions in siKlf4 as compared to siControl cells, while no change was observed with the tight junction protein ZO-1 (Figure 2C). Consistent with these findings, depletion of Klf4 expression in the presence of TGFβ markedly accelerated the EMT process; siKlf4 cells became more spindle-shaped and fibroblast-like as early as 2 days of TGFβ treatment, resembling siControl cells treated with TGFβ beyond 10 days (Figure 2A, middle and right panels). Immunoblotting and immunofluorescence analysis at day 2 of TGFβ treatment revealed that the morphological changes in siKlf4 cells were accompanied by increased expression of the mesenchymal markers vimentin and N-cadherin and an accelerated loss of the epithelial markers ZO-1 and E-cadherin from the cell membranes (Figure 2B, C). Notably, ablation of Klf4 expression in NMuMG cells that had been previously treated with TGFβ for 15 days still induced a more mesenchymal and spindle-like cell morphology as compared to siControl cells, indicating that Klf4 is not only inhibitory to the initiation but also to the maintenance of EMT (Figure 2A, right panel). The acceleration of EMT upon depletion of Klf4 function was further confirmed in Py2T murine breast cancer cells (Figure S2A–D). Together, these results demonstrate a critical role of Klf4 in maintaining epithelial morphology and in preventing EMT.

### Klf4 Prevents Cell Migration and Promotes Cell Survival during EMT

**Figure 3:**
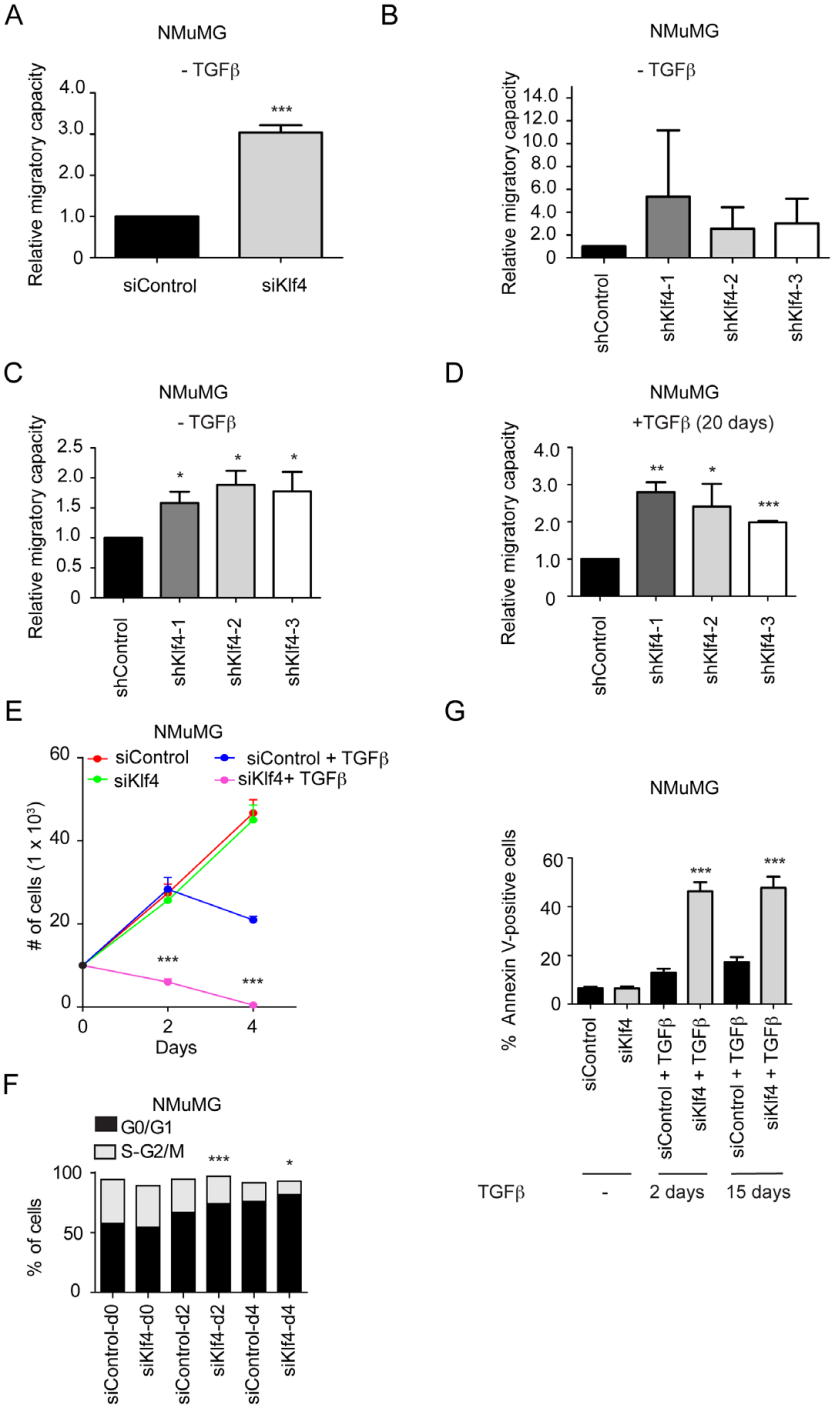
Klf4 inhibits cell migration and promotes cell survival and proliferation during TGFβ-induced EMT. (**A**) siRNA-mediated ablation of Klf4 results in an increase in NMuMG cell migration even in the absence of TGFβ as determined by scratch wound closure assays (see pictures in Figure S3A). (**B**) Stable shRNA-mediated depletion in NMuMG cells using three different shRNA sequences specific for Klf4 results in moderately increased cell migration as determined by scratch wound closure assays (see pictures in Figure S3B). (**C**) Stable shRNA-mediated depletion of Klf4 expression in NMuMG cells results in increased cell migration as determined by trans-well migration assays using 20% FBS as a chemo-attractant in the absence of TGFβ. (**D**) Stable shRNA-mediated depletion of Klf4 expression in NMuMG cells previously treated for 20 days with TGFβ results in increased cell migration as determined by trans-well migration assays using 20% FBS as a chemo-attractant. (**E**) siRNA-mediated ablation of Klf4 expression during TGFβ treatment of NMuMG cells results in a significant decrease in cell numbers as compared to cells transfected with control siRNA, while the depletion of Klf4 has no effect on cell number in the absence of TGFβ. (**F**) NMuMG cells transfected with either control siRNA (siControl) or with siRNA against Klf4 (siKlf4) were treated with TGFβ for the days indicated. Cells were stained with propidium iodide (PI), and the percentages of cells in G0/G1 and S-G2/M phases of the cell cycle were determined by flow cytometry analysis. (**G**) NMuMG cells transfected with either control siRNA (siControl) or with siRNA against Klf4 (siKlf4) were treated with TGFβ for the days indicated, and the rates of apoptosis were determined by Annexin-V staining and flow cytometry. Note that in the 15 days experiment, cells were first treated with TGFβ for 13 days and then transfected with siRNA constructs. Statistical values were calculated using an unpaired/paired, two-tailed t-test and experiments were performed at least three times. * = p≤0.05; ** = p≤0.01; *** = p≤0.001.

Since cell migration constitutes a key feature in forming metastasis via EMT, we assessed the migratory capacity of Klf4 knockdown cells. Scratch wound closure assays revealed that siKlf4 NMuMG cells migrated significantly faster than siControl cells even in the absence of TGFβ (Figure 3A and. Figure S3A). Scratch wound closure was also moderately accelerated in NMuMG cells stably depleted for Klf4 expression (shKlf4) by using three different shRNAs as compared to shControl cells (Figure 3B and Figure S3B). Moreover, transwell migration assays with shKlf4 cells revealed a significantly higher chemotactic migration as compared to shControl cells both in the absence and presence of TGFβ (Figure 3C and D, respectively). Comparable results were observed in Py2T cells depleted of Klf4 (Figure S3C). Together, these results indicate that Klf4 represses cell migration.

Klf4 has been shown to inhibit proliferation and promote differentiation of skin and colonic epithelium [14], [25]. Thus, we next investigated whether Klf4 depletion affected NMuMG cell proliferation and survival during TGFβ-induced EMT. Surprisingly, depletion of Klf4 expression led to a decrease in NMuMG cell number after treatment with TGFβ, an effect that was not observed in the absence of TGFβ (Figure 3E). Propidium iodide staining and flow cytometry analysis revealed a significant G0/G1 cell cycle arrest in siKlf4 cells as compared to siControl cells in the presence of TGFβ (Figure 3F). In addition, Annexin V staining showed an increase in apoptosis in siKlf4-transfected cells after treatment with TGFβ as compared to siControl-transfected cells (Figure 3G). Hence, the TGFβ-sensitive, decreased growth of siKlf4 cells depended on both decreased proliferation and increased apoptosis. Unlike NMuMG cells, Py2T cells did not undergo apoptosis during TGFβ-induced EMT. However, apoptosis in Klf4-depleted Py2T cells was significantly higher as compared to siControl Py2T cells, although not as substantial as observed with NMuMG cells (Figure S3D), and siKlf4 Py2T cells did not arrest in G0/G1 during TGFβ treatment (Figure S3E). Together, these results reveal an inhibitory role of Klf4 in cell migration as well as a supportive role in cell proliferation and cell survival during TGFβ-induced EMT, with varying degrees depending on the cells’ transformation status.

### Ectopic Expression of Klf4 Prevents EMT

**Figure 4:**
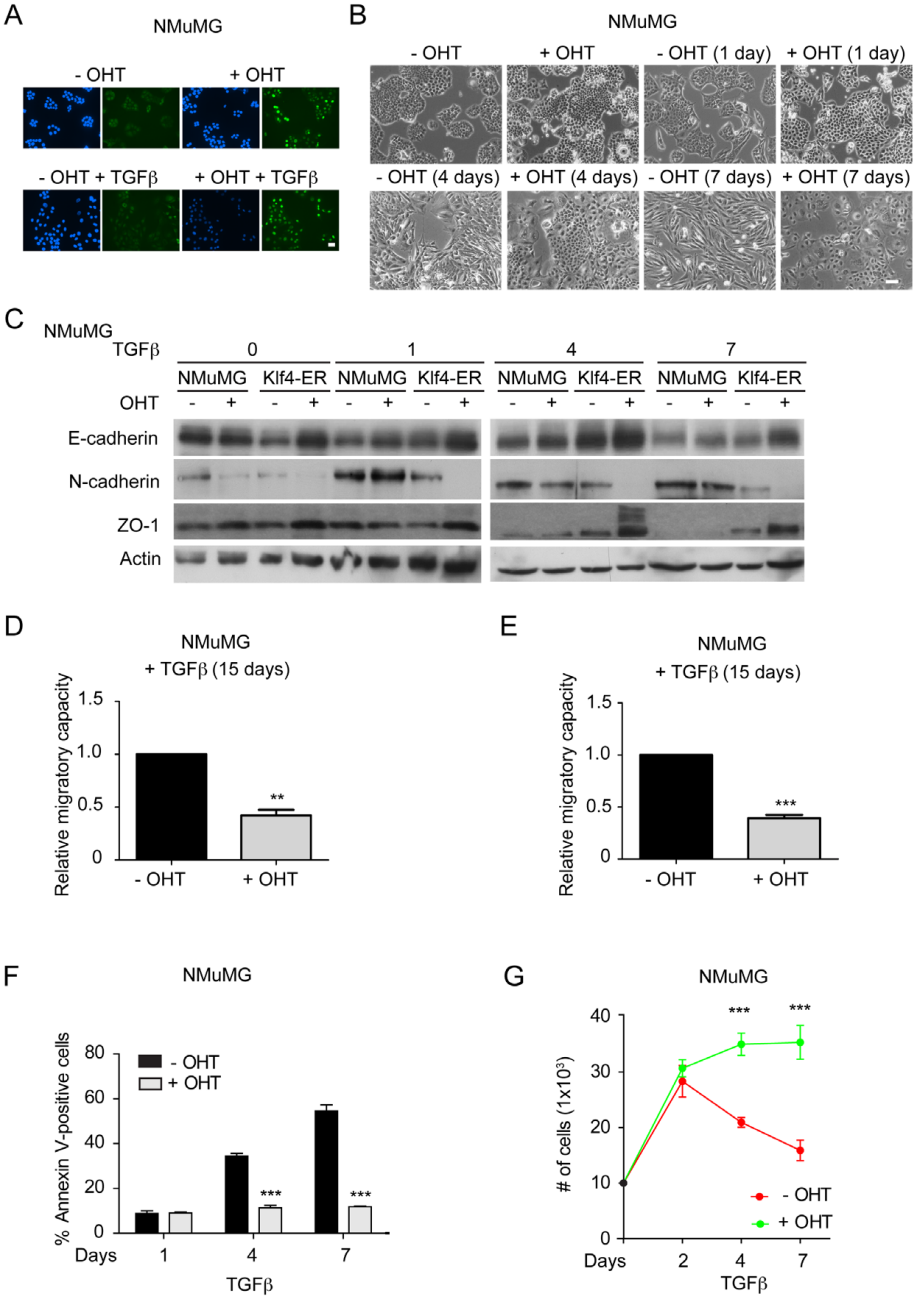
Klf4 prevents EMT and cell migration and supports cell survival during EMT. (**A**) Immunofluorescence staining of NMuMG cells stably expressing a Myc-Klf4-ER™ fusion protein using an anti-Myc-tag antibody to visualize the nuclear localization of Klf4 with and without 4-hydroxy-Tamoxifen (± OHT) treatment in the absence as well as in the presence of TGFβ for 2 days. Size bar, 50 µm. (**B**) Induction of Klf4 transcriptional activity by treatment of Myc-Klf4-ER™-expressing NMuMG cells treated with 4-OHT (+OHT) results in a repression of the morphological changes occurring during TGFβ-induced EMT in control-treated cells (−OHT). Shown are phase contrast images of Myc-Klf4-ER™ expressing NMuMG cells treated with TGFβ for 0, 1, 4 and 7 days in the absence or presence of 4-OHT. Size bar, 100 µm. (**C**) Immunoblotting analysis of the expression of the epithelial markers E-cadherin and ZO-1 and the mesenchymal marker N-cadherin during TGFβ-induced EMT in control NMuMG cells and in Myc-Klf4-ER™-expressing NMuMG cells in which Klf4 transcriptional activity has been induced (+OHT) or not (−OHT). Activation of Klf4 in Klf4-ER (+OHT) cells results in the maintenance of the expression of epithelial markers and the failure to express mesenchymal markers. Immunoblotting for actin was used as loading control. (**D**) Activation of Klf4 by treatment of Myc-Klf4-ER™-expressing NMuMG cells with 4-OHT (+OHT) results in reduced cell migration in scratch wound closure assays as compared to control-treated Myc-Klf4-ER™-expressing NMuMG cells (−OHT). Cells have been treated with TGFβ for 15 days prior to the migration assays. (**E**) Activation of Klf4 (+OHT) represses cell migration in trans-well migration assays by using 20% FBS as a chemoattractant for 20 hours in 15 days TGFβ-treated Myc-Klf4-ER™-expressing NMuMG cells. (**F**) Induction of Klf4 transcriptional activity by treatment of Myc-Klf4-ER™-expressing NMuMG cells with 4-OHT (+OHT) results in reduced apoptosis as compared to Myc-Klf4-ER™-expressing NMuMG cells in the absence of 4-OHT (−OHT). Apoptosis was quantified by Annexin-V staining and flow cytometry. (**G**) Activation of Klf4 by treatment of Myc-Klf4-ER™-expressing NMuMG cells with 4-OHT (+OHT) results in a significant increase in cell numbers as compared to the same cells cultured in the absence of 4-OHT (−OHT). Statistical values were calculated using an unpaired/paired, two-tailed t-test and experiments were performed at least three times. ** = p≤0.01; *** = p≤0.001.

We next assessed whether the forced expression of Klf4 was sufficient to prevent EMT. We generated NMuMG cells expressing a Myc-Klf4-estrogen receptor (ER™) fusion protein, where Klf4-ER™ translocated to the nucleus upon treatment with the Tamoxifen derivative, 4-hydoxy-Tamoxifen (4-OHT [19]; Figure 4A). Upon treatment of the cells with 4-OHT, morphological changes in the presence and absence of TGFβ were determined. Interestingly, despite the presence of TGFβ, Klf4-activated cells largely retained their epithelial phenotype (+OHT), also manifested by the high expression of the epithelial markers E-cadherin and ZO-1 and a failure to induce expression of mesenchymal markers, such as N-cadherin, compared to control-treated cells (−OHT; Figure 4B and C). Klf4 also inhibited cell migration in scratch wound closure and transwell migration assays (Figure 4D and 4E). In addition, Klf4 activation also blocked TGFβ-induced apoptosis (Figure 4F) and, as a result, cell numbers were increased in growth curves as compared to Klf4-ER™-transfected NMuMG cells in the absence of 4-OHT (Figure 4G). Comparable results were obtained with Klf4-ER™-transfected Py2T cells, where the 4-OHT-mediated activation of Klf4 attenuated TGFβ-induced EMT (Figure S4A and B). These results suggested that Klf4 elicits a transcriptional program that is critical in the maintenance of the epithelial phenotype and the prevention of EMT.

### Klf4 Blocks Malignant Tumor Progression and Metastasis

**Figure 5:**
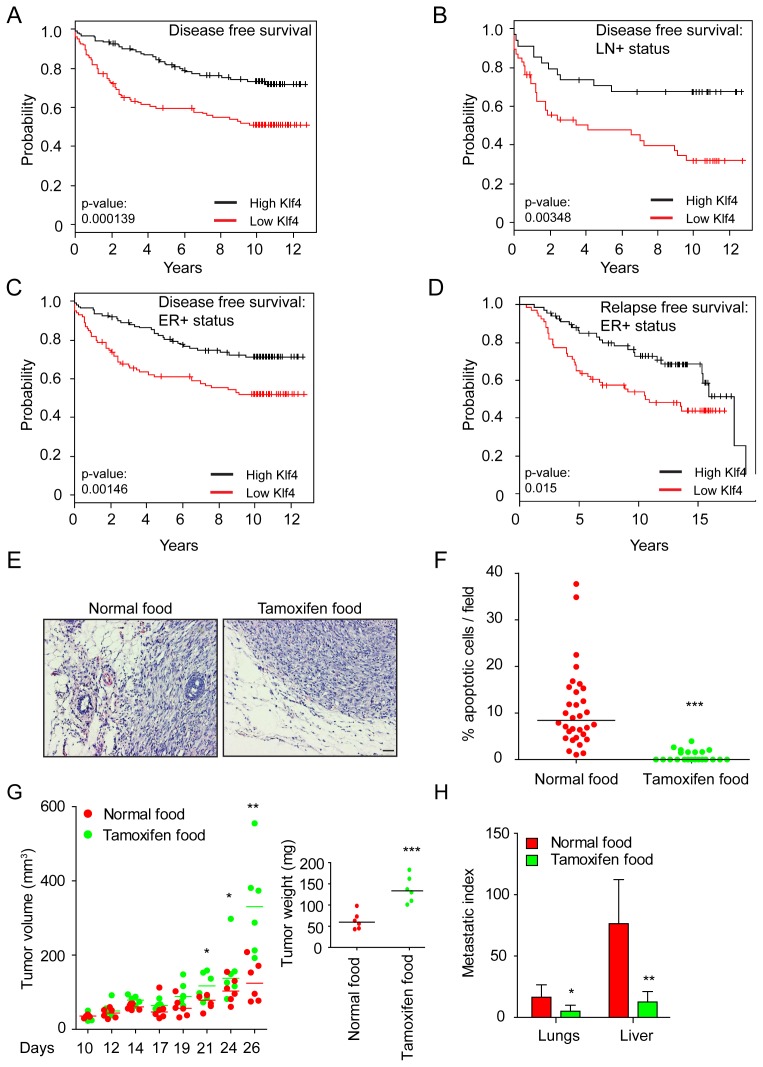
High Klf4 expression correlates with good survival prognosis in patients and prevents metastasis in experimental models. (**A–C**) Kaplan-Meier survival analysis reveals a significant correlation between low Klf4 expression levels and poor overall disease-free survival (A) by correlating high or low Klf4 expression levels with all patient samples (Uppsala database). Correlation of Klf4 expression levels with disease-free survival was further determined after stratifying the patient samples into lymph node-positive (LN+; B) and estrogen receptor-positive (ER+; C) tumor groups. (**D**) Kaplan-Meier survival analysis reveals a significant correlation between poor relapse-free survival in ER+ breast cancer patients (TRANSBIG database) and low Klf4 expression. (**E**) Myc-Klf4-ER™ and firefly luciferase-expressing Py2T cells were orthotopically transplanted into the mammary fat pad of immune-compromised Balb/c nude mice. Mice were treated with normal food or with food containing Tamoxifen to induce Klf4 transcriptional activity. Shown are images of histological sections of primary tumors stained with hematoxylin and eosin. Note that Klf4 appears to repress the invasive morphology of the tumors. Size bar, 100 µm. (**F**) The percentages of apoptotic cells were quantified by cleaved caspase 3 staining of histological tumor sections described in (E). Activation of Klf4 by Tamoxifen repressed apoptosis in the primary tumors. (**G**) Primary tumor volumes were calculated after transplantation of Myc-Klf4-ER™ cells as described in (E) while tumor weights were calculated after sacrificing the tumor-bearing mice 27 days post-injection. Activation of Klf4 by Tamoxifen promoted tumor growth. (**H**) Metastatic spread was determined in the Myc-Klf4-ER™ cells -transplanted mice described in (E) by measuring luciferase activity in extracts of lungs and livers of the transplanted mice. Luciferase activity levels in the various organs were divided by the primary tumor weights within the same mice to establish the metastatic index, as shown. Activation of Klf4 by Tamoxifen represses metastatic spread of the transplanted tumor cells. Statistical values for panels F – H were calculated using an unpaired/paired, two-tailed t-test. * = p≤0.05; ** = p≤0.01; *** = p≤0.001.

We next assessed whether decreased expression of Klf4 correlated with the ability of breast cancer to progress and metastasize in patients. A positive and significant correlation of decreased Klf4 expression with tumor grade has been previously reported [21]. Our analysis of the Uppsala database of breast cancer [29], [30] revealed that low Klf4 expression correlated with shorter disease-free survival. This correlation was highly significant with breast cancers expressing estrogen receptor (ER+) or exhibiting lymph node metastasis (LN+) (Figure 5A–C) and was lower but still significant in breast cancers with ER− and LN− status (Figure S5A and B). Moreover, in the TRANSBIG breast cancer database [31], low Klf4 expression levels correlated with shorter relapse-free survival of ER+ breast cancer patients (Figure 5D).

These observations led us to investigate whether the forced expression of Klf4 in Py2T cells could affect the ability of these cells to form tumors and undergo metastasis upon transplantation into mice. We generated inducible Klf4-expressing Py2T cells (Klf4-ER™) that also expressed firefly luciferase and showed high Klf4 expression and nuclear localization in the presence of 4-OHT (Figure S5C). These cells were then orthotopically implanted into the mammary fat pad of nude mice, and tumor growth and metastasis in lung and liver were quantified in mice treated or not with Tamoxifen in the food. Histopathological examination of the primary tumors revealed an invasive and dedifferentiated phenotype in the absence of Tamoxifen and a more differentiated and less invasive morphology upon Tamoxifen treatment (Figure 5E). In addition, expression of epithelial marker E-cadherin is retained at the cell membrane while expression of mesenchymal markers N-cadherin and fibronectin are decreased after Klf4 induction in tumor sections (Figure S5D). Similar to the findings *in vitro*, the activation of Klf4 in Py2T cells led to significantly decreased rates of apoptosis in Klf4-activated tumors (Figure 5F), while tumor cell proliferation was only moderately increased (Figure S5E). As a consequence, tumor growth and tumor weight were significantly increased upon activation of Klf4 (Figure 5G), yet decreased luciferase levels were observed in organs that are known targets of metastasizing breast cancer cells, such as lung and liver (metastatic index; Figure 5H; Figure S5F). Together, these results indicate that Klf4 activity increases primary breast tumor growth but prevents its metastatic spread.

### Klf4 Directly Regulates the Expression of Crucial EMT Genes

**Figure 6:**
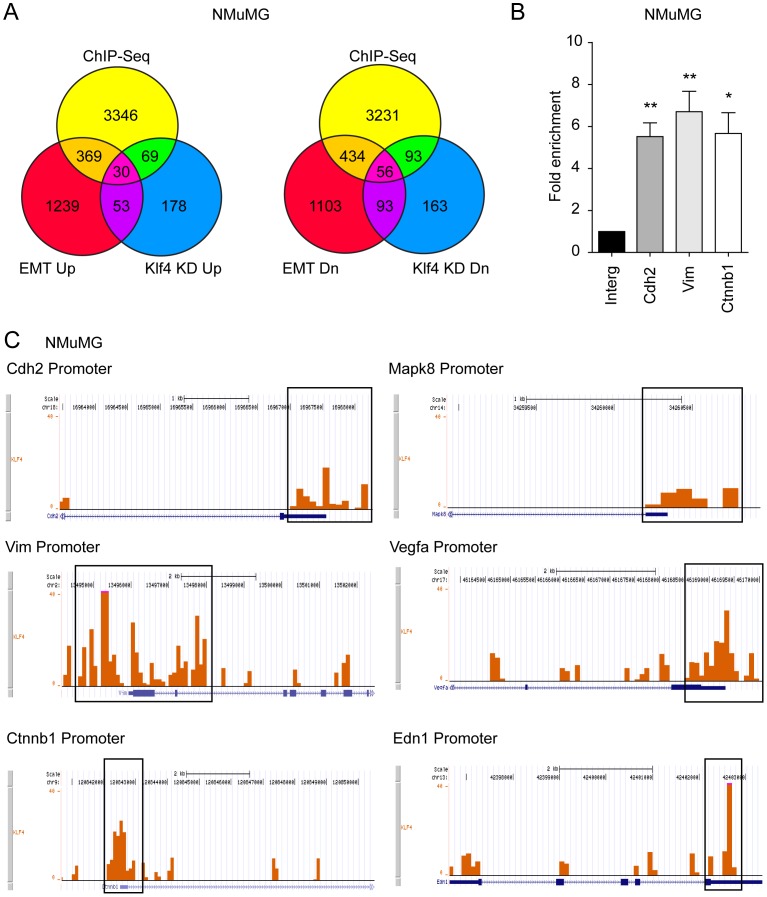
Klf4 directly regulates the expression of crucial EMT genes. (**A**) Venn-diagrams depicting the commonly regulated genes between the data obtained by Klf4 chromatin immunoprecipitation-deep sequencing (ChIP-Seq), gene expression profiling of TGFβ-induced EMT (EMT Up and Dn, respectively), and gene expression profiling of siRNA-mediated Klf4 depletion in the presence of TGFβ for 2 days (Klf4 Up and Dn, respectively), all performed in NMuMG cells. The numbers of genes directly up-regulated (left diagram; 30 genes) or down-regulated (right diagram; 56) during EMT are shown (see Table S1 for the gene names). (**B**) Chromatin immunoprecipitation with antibody against Klf4 followed by quantitative PCR (ChIP-qPCR) to demonstrate the occupancy of Klf4 at the promoters of the N-cadherin (*Cdh2*), vimentin (*Vim*) and β-catenin (*Ctnnb1*) genes. The qPCR data were normalized to ChIP-qPCR of an intergenic region. (**C**) Wiggle-tracks to show the binding of Klf4 at the promoters of the N-cadherin (*Cdh2*), vimentin (*Vim*), β-catenin (*Ctnnb1*), Jnk1 (*Mapk8*), vascular endothelial growth factor A (*Vegfa*) and endothelin1 (*Edn1*) genes by using UCSC genome browser. These files were generated from Klf4 ChIP-Seq data of NMuMG cells. Statistical values were calculated using an unpaired/paired, two-tailed t-test and experiments were performed at least three times. * = p≤0.05; ** = p≤0.01.

Our observations propose a critical transcriptional role of Klf4 in the regulation of primary tumor growth, EMT and metastasis. Hence, we set out to identify the genes that are regulated by Klf4. Genome-wide gene expression profiling between NMuMG cells either transfected with control siRNA (siControl) or with siRNA against Klf4 (siKlf4) in the absence and presence of TGFβ (2 days) revealed a number of genes that were significantly up and down-regulated in response to Klf4 expression. To identify the direct target genes of Klf4, i.e. the genes whose promoters are directly bound by Klf4, we performed chromatin immunoprecipitation using a Klf4-specific antibody in NMuMG cells followed by next generation sequencing (ChIP-Seq). The list of gene promoters directly bound by Klf4 was compared to the lists of genes that were changed in their expression in dependence on Klf4 and that were regulated during the different stages of TGFβ-induced EMT in NMuMG cells (Figure 6A; Figure S6A-D). This analysis identified 33 direct Klf4 target genes with increased expression during EMT and 57 direct Klf4 target genes with reduced expression during EMT (Table S1). Gene ontology analysis revealed that the direct target genes of Klf4 are mainly active in signaling pathways regulating EMT, cell adhesion, cell cycle control and angiogenesis (Table S2). Among the genes, we identified a number of well-established EMT marker genes, including the epithelial marker gene E-cadherin (*Cdh1*), previously shown to be directly activated by Klf4 [26], and the mesenchymal markers N-cadherin (*Cdh2*), vimentin (*Vim*), β-catenin (*Ctnnb1*), vascular endothelial growth factor A (*Vegfa*) and endothelin-1 (*Edn1*), all of which are up-regulated in their expression upon loss of Klf4 function during EMT. Quantitative real-time RT-PCR showed that expression of these genes was in fact modulated by Klf4 activity (data not shown). ChIP followed by quantitative PCR using specific primers for the particular gene promoters confirmed the direct binding of Klf4 to the promoters of the N-cadherin (*Cdh2*), vimentin (*Vim*) and β-catenin (*Ctnnb1*) genes (Figure 6B), and the DNA fragments bound by Klf4 were found within the promoter regions close to the transcriptional start sites of these genes (Figure 6C). The genes encoding for N-cadherin, vimentin and β-catenin were also confirmed as direct transcriptional targets of Klf4 in Py2T cells by ChIP-qPCR (Figure S6E). These results indicate that Klf4 is a transcriptional activator of epithelial genes and a transcriptional repressor of mesenchymal genes and that its loss represents a major switch in the initiation of EMT.

### Klf4 Prevents EMT by Repressing Jnk1 Expression

**Figure 7:**
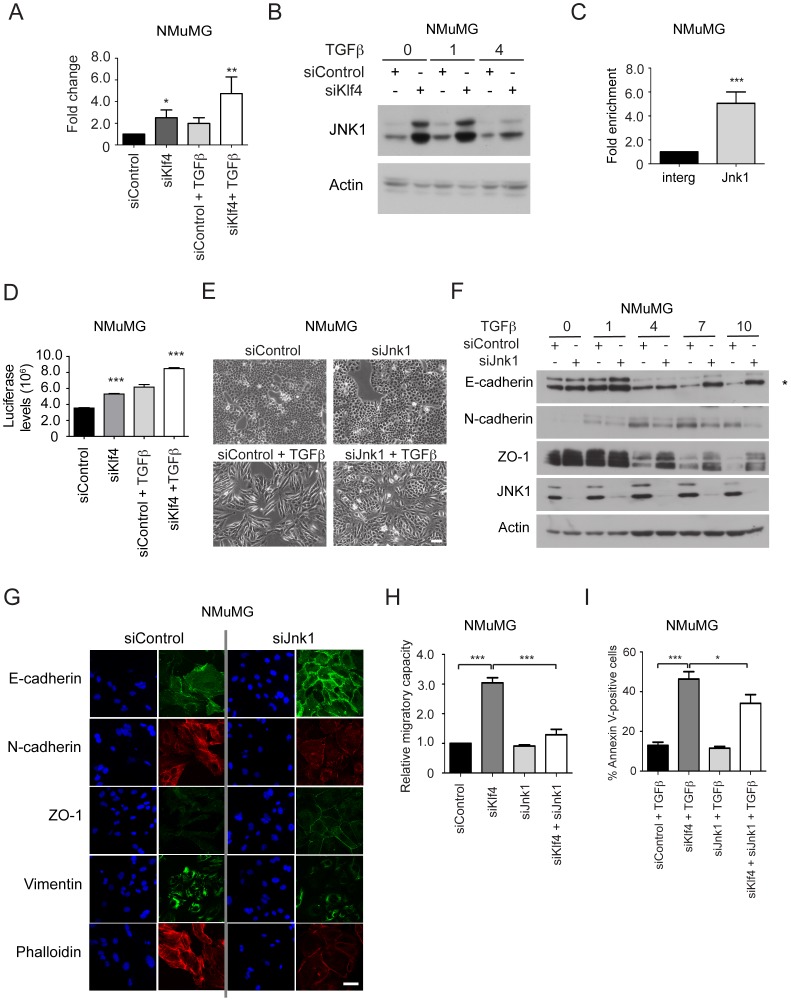
Klf4 directly represses Jnk1 (*Mapk8*) gene expression, and Jnk1 is required for TGFβ-induced EMT and loss of Klf4-induced cell migration and apoptosis in NMuMG cells. (**A**) siRNA-mediated depletion of Klf4 expression increases Jnk1 expression as determined by quantitative RT-PCR. NMuMG cells were treated or not with TGFβ for 2 days. (**B**) Ablation of Klf4 function induces Jnk1 expression. Immunoblotting analysis of the expression of Jnk1 during TGFβ-induced EMT in NMuMG cells transfected with control siRNA (siControl) or with siRNA against Klf4 (siKlf4). Immunoblotting for actin was used as loading control. (**C**) Klf4 directly binds the Jnk1 (*Mapk8*) gene promoter in NMuMG cells as determined by ChIP using an antibody against Klf4 followed by qPCR using primers specific for the promoter region of the Jnk1 (*Mapk8*) gene. The qPCR data were normalized to ChIP-qPCR of an intergenic region. (**D**) An AP1 activity reporter assay was performed to assess Jnk1/AP-1-mediated transcriptional activity after Klf4 depletion (siKlf4) in NMuMG cells with or without TGFβ for 2 days. *Firefly* luciferase activity was normalized to co-transfected *Renilla* luciferase activity (relative luminescence). (**E**) Ablation of Jnk1 expression prevents EMT. Phase contrast microscopy of NMuMG cells transfected with control siRNA (siControl) and siRNA against Jnk1 (siJnk1) before and after TGFβ treatment for 2 days. Size bar, 100 µm. (**F**) Jnk1 depletion represses EMT. Immunoblotting analysis of the expression levels of Jnk1, the epithelial markers E-cadherin (the correct band is marked with an asterisk) and ZO-1 and the mesenchymal marker N-cadherin in NMuMG cells treated with either control (siControl) or Jnk1-specific siRNA (siJnk1) during TGFβ-induced EMT. Immunoblotting for Jnk1 was used to assess the siRNA-mediated knock-down efficiency, and immunblotting for actin was used as a loading control. (**G**) Down-regulation of Jnk1 expression prevents EMT. The localization and expression levels of EMT markers as indicated was assessed by immunofluorescence microscopy after 7 days of TGFβ treatment in siControl and siJnk1-transfected NMuMG cells. Size bar, 50 µm. (**H**) Jnk1 is required for cell migration of NMuMG cells induced by the loss of Klf4 expression, as determined by scratch wound closure assays of control (siControl), Klf4-depleted (siKlf4), Jnk1-depleted (siJnk1), or siKlf4 and siJnk1 double-depleted (siKlf4+siJnk1) NMuMG cells. (**I**) Jnk1 is required for apoptosis of NMuMG cells induced by the loss of Klf4 expression and TGFβ treatment for 2 days, as determined by Annexin V staining and flow cytometry of control (siControl), Klf4-depleted (siKlf4), Jnk1-depleted (siJnk1), or siKlf4 and siJnk1 double-depleted (siKlf4+siJnk1) NMuMG cells. Statistical values were calculated using an unpaired/paired, two-tailed t-test and experiments were performed at least three times. * = p≤0.05; ** = p≤0.01; *** = p≤0.001.

Among the various genes identified in the course of our experiments, Jnk1 (*Mapk8*) was significantly up-regulated in its expression at the mRNA and protein level in Klf4-depleted NMuMG (Figure 7A and B) and Py2T cells (Figure S7A) and down-regulated in Klf4 over-expressing NMuMG and Py2T cells (Figure S7B and C). ChIP using Klf4-specific antibody followed by qPCR revealed that Klf4 directly bound the Jnk1 gene promoter in NMuMG (Figure 7C) and Py2T cells (Figure S7D). Moreover, increased AP1 reporter activity after Klf4 depletion in the absence and presence of TGFβ confirmed the repressive role of Klf4 for Jnk1-mediated signal transduction (Figure 7D).

We next assessed the functional contribution of Jnk1 expression to EMT. Transient knockdown of Jnk1 using a pool of two siRNA (siJnk1) led to a significant reduction in Jnk1 expression (Figure S7E). Interestingly, similar to cells over-expressing Klf4, Jnk1 depletion preserved an epithelial differentiation status and lead to a retention of epithelial morphology even in the presence of TGFβ in NMuMG cells (Figure 7E). Immunoblotting and immunofluorescence analysis revealed that Jnk1-depleted cells maintained the expression of epithelial markers and failed to induce the expression of mesenchymal markers during TGFβ-induced EMT (Figure 7F, G). Moreover, TGFβ-induced stress fiber formation was also prevented (Figure 7G). Notably, while siRNA-mediated depletion of Klf4 induced EMT, cell migration and apoptosis, the depletion of Jnk1 in the Klf4-knockdown cells largely prevented these processes (Figure 7H, I; Figure S7F). These experiments demonstrate a critical role of Klf4 in preventing EMT and apoptosis by the direct transcriptional repression of the Jnk1 (*Mapk8*) gene, which itself is required for EMT, cell migration and TGFβ-induced apoptosis. However, the forced expression of Jnk1 in NMuMG cells was not sufficient to induce EMT in the absence of TGFβ or a reduced expression of Klf4 (data not shown), indicating that the loss of Klf4 expression and the changed expression of other Klf4 target genes are required for EMT to occur.

## Discussion

EMT is a multistage process that is essential in early embryonic development, a program that is hijacked by various pathological conditions, such as tissue fibrosis and malignant tumor progression and metastasis. EMT is accompanied by major alterations in cell morphology, cell-cell and cell-matrix adhesions and in the motile and invasive capabilities of cells, accomplished by major changes in gene expression during the acquisition and maintenance of an EMT. Here, using genome-wide gene expression profiling, we have identified Klf4 as one critical regulator of EMT. Notably, Klf4 is a major repressor of EMT: its expression prevents EMT and maintains an epithelial morphology of mammary epithelial cells and of breast cancer cells, while its ablation results in a loss of epithelial morphology and in a partial induction and acceleration of EMT. In addition, we find that Klf4 also supports cell proliferation and survival while inhibiting cell migration. This notion contrasts previous studies showing that Klf4 inhibits cell proliferation in a colon cancer cell line and that it acts as a pro-migratory factor in the MCF7 and MDA-MB-231 human breast cancer cell lines [32], [33]. These differences could be the result of higher levels of Klf4 in breast cancer cell lines. However, in contrast to the findings in the above study, we found downregulation of Klf4 in MCF7 cells after E-cadherin depletion and a recent report also demonstrated a migration and invasion-repressive role of Klf4 in MDA-MB-231 cells [26].

Klf4 is known for its dual role as a transcriptional activator and repressor. Previous studies have suggested that Klf4 is a critical factor in maintaining the cell morphology of mammary epithelial cells. It directly represses the expression of Snail1, one of the major transcriptional repressors of E-cadherin gene expression, and also directly binds to the E-cadherin gene promoter and up-regulates E-cadherin expression [26], [34]. Our combined genome-wide gene expression profiling and ChIP-Seq data analysis has also revealed Klf4 binding to the Snail1 (*Snai1*) and E-cadherin (*Cdh1*) gene promoters. Indeed, *Snai1* expression appears regulated by Klf4, as it is significantly up-regulated upon depletion of Klf4 in the absence of TGFβ. However, in the presence of TGFβ Snail1 expression is reduced in Klf4 knockdown cells (Figure S8A), suggesting that Klf4-mediated regulation of the *Snai1* gene is a complex process and besides Klf4 involves other transcription factors that repress or activate *Snai1* transcription. On the other hand, Klf4 ablation leads to an up-regulation of Twist1 expression in the presence of TGFβ that may act as the main driver of EMT in the mesenchymal state (Figure S8B). Reduced Klf4 expression did not alter the expression of Snail2, Zeb1, Zeb2 and other major EMT regulators (data not shown). Moreover, we identified additional genes that are directly induced or repressed in their expression by Klf4. Notably, a number of mesenchymal genes, such as N-cadherin (*Cdh2*), vimentin (*Vim*), and β-catenin (*Ctnnb1*), are direct targets of Klf4 transcriptional repression. Together, the data proposes a model in which Klf4 maintains epithelial morphology by inducing the expression of epithelial-specific genes and by repressing mesenchymal genes (Figure S9). Upon loss of Klf4 function during EMT, epithelial gene expression is lost, and mesenchymal genes are activated. A similar function of Klf4 was observed during reprogramming of fibroblasts into induced pluripotent stem (iPS) cells, where Klf4 as one of the key reprogramming factors activates *Cdh1* expression, resulting in mesenchymal-epithelial transition (MET) and reprogramming of fibroblasts [35]. These observations further illustrate that Klf4-mediated transcription control is not only important for maintaining the identity of epithelial cells but also in its establishment. Moreover, Klf4 also represses several genes involved in angiogenesis, including VEGF-A (*Vegfa*) and Endothelin-1 (*Edn1*). These results suggest that the loss of Klf4 expression during EMT is a critical event in promoting tumor angiogenesis, malignant tumor progression and metastasis.

We further show that one of the transcriptionally repressed target genes of Klf4 is Jnk1 (*Mapk8*); upon loss of Klf4 during TGFβ-induced EMT, Jnk1 is induced in its expression and exerts a critical function in the execution of EMT and cell migration but also in the induction of apoptosis. Jnk1 is a member of the mitogen-activated protein kinases (Mapk) family of serine-threonine protein kinases that participate in various signaling pathways and are critical for cellular responses to extracellular stimuli. Jnk1 is activated primarily by cytokines and cellular stress, such as UV light, which leads to Jnk1 phosphorylation at serine-63 and serine-73 residues [36]. Such activation of Jnk is required for UV-induced apoptosis in primary murine embryonic fibroblasts and to maintain the mitochondrial apoptosis signaling pathway [37]. Jnk1 and their target transcription factor AP1 (Jun/Fos) have also been previously implicated in EMT [38], [39], [40]. In line with these observations, Jnk1 can phosphorylate paxillin, a focal adhesion adaptor, required for the formation of focal adhesion plaques and for efficient cell migration [41]. Here, we show that Jnk1 exerts a critical role in EMT, cell migration and in the induction of apoptosis upon loss of its direct transcriptional repressor Klf4. Recent reports have demonstrated that Jnk1, Jnk2 or composite Jnk1 and 2 deficiencies promote primary tumor formation in various mouse models of breast cancer [42], [43], [44] These data are consistent with our findings that Klf4, by repressing Jnk1 gene expression, prevents apoptosis and promotes primary tumor growth while inhibiting metastasis formation.

Thus, our experimental data in cultured cells and in mouse transplantation models highlight Klf4’s function as a suppressor of malignant breast carcinogenesis. Moreover, we find that high Klf4 expression correlates with increased disease-free survival in patients, for example in the Uppsala breast cancer database [29], [30] and in the TRANSBIG breast cancer database [31]. However, our experimental results also reveal that Klf4 not only prevents lung and liver metastasis but also provides a survival advantage to cells, thus leading to increased primary tumor growth. These data are in contrast to findings reported by Yori et al. showing that the forced expression of Klf4 in 4T1 cells murine breast cancer cells not only reduces lung and liver metastasis but also primary tumor growth [34]. However, in another study these authors also observe a role of Klf4 in promoting cell survival [26], and Yu et al. have previously reported that silencing of Klf4 in MDA-MB-231 cells represses primary tumor growth [33].

Together, the data reported herein reveal fundamental principles of how transcription factors like Klf4 regulate cell-fate changes by directly modulating the transcription of underlying genes. In addition to demonstrating that Smad4-dependent TGFβ signaling represses Klf4 expression, our data also suggest that Wnt and EGFR signaling may also contribute to Klf4 transcriptional regulation, as treatment of NMuMG and Py2T cells with the Wnt activator LiCl or with an inhibitor of EGFR signaling (EGFRi) leads to up-regulation of Klf4 in the absence as well as in the presence of TGFβ (Figure S1C and D). However, the detailed molecular mechanisms of this regulation during EMT remain to be delineated. Finally, the critical functions of Klf4 in the maintenance of epithelial differentiation and in the prevention of EMT and metastasis make Klf4 and its transcriptional effectors attractive targets for the development of novel therapeutic approaches. Notably, the activities of Jnk1 that are up-regulated upon loss of Klf4 function may offer a therapeutic avenue to metastatic disease, a notion that warrants further investigation.

## Materials and Methods

### Reagents and Drugs

#### Reagents

PBS (D8537, Sigma-Aldrich), trypsin (T4174, Sigma-Aldrich), Opti-MEM (11058, Gibco), Glutamine (G7513, Sigma-Aldrich), Pencillin/streptomycin (P4333, Sigma-Aldrich), Alexa Fluor-488, 568, 633 (Invitrogen), Polybrene (AL-118, Sigma-Aldrich), Fugene HD (12998300, Roche), Bradford reagent (500-0006, Biorad), Protease inhibitor cocktail (P2714, Sigma-Aldrich).

*Drugs:* TGFβRi (SB431542; S4317, Sigma); EGFRi (AG1478; ALX-270-036, Axxora); LiCl (L9650, Sigma).

### Cell Lines and Cell Culture

A subclone of NMuMG cells (NMuMG/E9; hereafter NMuMG) [45] and MCF7 shControl and MCF7-shEcad have been described before [9]. Py2T cells were isolated from a breast tumor of an MMTV-PyMT transgenic female mouse in an FVB/N background [27]. EpRas cells have been obtained from H. Beug (IMP, Vienna) and SKBR3 cells have been obtained from ATCC. NMuMG, Py2T and EpRas cells were cultured in DMEM supplemented with glutamine, penicillin, streptomycin, and 10% FBS (Sigma) while SKBR3 cells were grown in RPMI-1640 supplemented with glutamine, penicillin, streptomycin and 10% FBS. NMuMG-shSmad4 and NMuMG-shControl were kindly provided by P. ten Dijke (LUMC, The Netherlands) [28]. Cells were cultured in DMEM (D5671, Sigma)/10% FBS (F7524, Sigma) and treated with 2 ng/ml TGFβ (240-B, R&D systems) for the indicated time points and replaced every 2nd day. For siRNA transfections, Lipofectamine RNAiMax (11668-019, Invitrogen) was used according to the manufacturer's instructions.

### Cell Growth

For growth curves, cells were counted as described earlier [8]. For Cell cycle analysis, propidium iodide (PI; P4170, Sigma) was used according to the manufacturer’s instructions. Stained cells were analyzed on a FACSCanto II using DIVA software.

### Production of Lentivirus for Knockdown Studies and Retrovirus for Overexpression Studies

Murine Klf4 shRNAs and control shRNA were used for knockdown studies while Myc-Klf4-ER construct (kindly provided by Prof. J.M. Ruppert), cloned into the retroviral expression vector pBabe, was used for overexpression studies. Lentivirus production and retroviral production have been previously described [8], [46]. After viral production, viral supernatant was filtered (0.46 µm) and target cells were transduced. Infected cells were positively selected using puromycin (5 ug/ml).

### Scratch Wound Closure Assay

In vitro wound healing assays were performed on confluent cells transfected with siControl (Stealth RNAi™ siRNA Negative Controls, 12935-100, Invitrogen), siKlf4 (SASI_Mm01_00114982 and SASI_Mm01_00114983, Sigma), siJnk1 (SASI_Mm01_00163536 & SASI_Mm01_00163537, Sigma) and siKlf4+ siJnk1 as previously described [47]. Briefly, the media of the confluent cells was replaced with DMEM containing 2% fetal bovine serum media and an area was scraped off using a 200 µl pipette tip. Light microscopic images were taken at time 0 and at 19 hours and the derived data was further analyzed using ImageJ software to quantify closed area after 19 hours compared to 0 hour.

### Migration Assay

Cell migration was assessed in shControl (Mission Non-target shRNA control vector, SHC002, Sigma) and shKlf4 (SHCLNG-NM_010637 Mouse, TRCN0000095370; TRCN0000095371 and TRCN0000095372, Sigma) as described previously [9]. Pictures of the membrane were taken at a 10× magnification using a fluorescent microscope (Nikon Diaphot 300). Quantification was done using ImageJ software.

### Apoptosis Assay (Annexin V Assay)

Annexin V antibody was purchased from BD Biosciences (559934) and staining was performed according to manufacturer’s instruction. Stained cells were filtered through 40 µm mesh and analyzed on a FACSCanto II using DIVA software.

### Quantitative RT-PCR

Total RNA isolation, cDNA synthesis and quantitative RT-PCR were performed as described previously [9]. Primer sequences are available upon request.

### Immunoblotting

Immunoblotting was performed as described previously [9] using following antibodies: Klf4 (09–821, Millipore), E-Cadherin (610182, Transduction Laboratories), N-Cadherin (M142, Takara), ZO-1 (617300, Zymed), Fibronectin (F-3648, Sigma), ZO-1 (617300, Zymed), Vimentin (V2258, Sigma), Vinculin (SC-7649, Santa Cruz Biotechnology), Jnk1/3 (SC-474, Santa Cruz), GAPDH (G8795, Sigma or ab9485, Abcam for E9 and Py2T lysates, respectively) and Actin (SC-1616, Santa Cruz).

### Immunofluorescence

Cells were plated on coverslips and treated with TGFβ for the times indicated. Immunofluorescence was performed as described before [9] using following antibodies: E-Cadherin (13–1900, Zymed), N-Cadherin (610921, Transduction Laboratories), Fibronectin (F-3648), ZO-1 (617300, Zymed), Phalloidin-568 (A12380, Invitrogen), Paxillin (13520, Transduction Laboratories), Vimentin (V2258, Sigma) and anti-Myc-tag (a gift by M. Cabrita). The coverslips were counterstained with DAPI (D9542, Sigma) and imaged with a Zeiss Meta 510 confocal microscope.

### Tumor Transplantations

1×10^6^ Myc-Klf4-ER™ and firefly luciferase-expressing Py2T cells in 100 µl of PBS were injected orthotopically into the mammary fat pad of 7 weeks old female Balb/c nude mice. Mice were kept on either normal food or Tamoxifen food (TD.55125, Harlan Laboratories, Inc.). After 27 days of treatment, mice were sacrificed, and primary tumors, lungs and livers were isolated. Luciferase levels were measured in lysates prepared from lungs and liver to to detect metastasis and to calculate the metastatic index by dividing the average luciferase levels in each organ with average tumor weights in the mice. All experimental procedures involving mice were performed according to the guidelines of the Swiss Federal Veterinary Office (SFVO) and the regulations of the Cantonal Veterinary Office of Basel Stadt.

### Histological Analysis

Paraffin and OCT sections were prepared as described previously [9]. Tumor cell proliferation and apoptosis was determined by staining frozen tumor sections for cleaved caspase-3 (9664, Cell Signaling) and for phospho-Histone 3 (06–570, Upstate), respectively. Subsequent quantification was performed using Image J software, and the results are displayed as percentage of the area of positively stained cells compared to the total tumor area in the microscopic field.

### Microarray Processing and Data Analysis

The manufacturer’s protocols for the GeneChip platform by Affymetrix were followed for microarray experiment in siControl and siKlf4 transfected NMuMG cells in the absence and presence of TGFβ. Raw microarray data were normalized with Robust Multi-Array (RMA) and analyzed using X-RayProj Software. One-way analysis of variance (ANOVA) and asymptotic analysis were used to identify significantly differentially expressed genes. The microarray data has been deposited on ArrayExpress platform under the accession number xxxx.

### Chromatin Immunoprecipitation

ChIP experiments were performed as previously described [8], [48], starting with 70 µg of chromatin and 5 µg of anti-Klf4 (SC-20691, Santa Cruz). Quantitative PCR after ChIP was performed using SYBR Green, using 1/40 of the ChIP sample or a 1∶100 dilution of input DNA per PCR reaction. Primer sequences are available upon request.

### ChIP-Seq Data Analysis

The ChIP libraries were prepared with the Illumina ChIP-Seq DNA Sample Prep Kit (Cat# IP-102–1001) according to Illumina’s instructions and sequenced on the Genome Analyzer 2 following the manufacturer’s protocols. Subsequent analysis was performed as described previously [49].

### Kaplan-Meier Survival Analysis

Kaplan-Meier survival analysis was performed on patients from the Uppsala and TRANSBIG cohorts [30], [31]. Based on the median expression of Klf4 (probeset 221841_s_at on HGU133A Affymetrix chip), the tumors of the Uppsala and TRANSBIG cohorts were divided into a high and a low Klf4 group. Kaplan-Meier survival analysis and Cox proportional hazards regression modeling was performed with the survival package 2.36–5 of R version 2.11.1 (www.r-project.org). The p-value of the likelihood-ratio test was calculated between the different groups and considered statistically significant below 0.05.

### AP1 Activity Reporter Assay

NMuMG cells overexpressing the AP1 luciferase reporter construct (SA Biosciences) were plated in triplicate in a 24 well plate and reversely transfected with Klf4 siRNAs. One day after plating, cells were transfected with renilla expressing plasmid. Fresh growth medium was added after 5 hours of transfection containing 2 ng/mL TGFβ or not. Two days after, cells were lysed using 1× passive lysis buffer (#E194, Promega), and lysates were analyzed using the Dual-Luciferase Reporter Assay System (#E1960, Promega) and a Berthold Luminometer LB960.

### Statistical Analysis

Statistical analysis and graphs were generated using the GraphPad Prism software (GraphPad Software Inc, San Diego, CA). All statistical analysis was done by unpaired/paired, two-sided t-test.

### Ethics Statement

Animal experiments were performed in strict accordance with the guidelines of the Swiss Federal Veterinary Office (SFVO) and the regulations of the Cantonal Veterinary Office of Basel-Stadt (license numbers 1878, 1907, and 1908). During the whole course of animal experiments, all efforts were made to minimize suffering.

## Supporting Information

Figure S1 **Klf4 expression is repressed by canonical TGFβ signaling during TGFβ-induced EMT in NMuMG cells. (A)** Morphological changes observed in NMuMG cells treated with TGFβ for 0, 1, 4, 7, 10 and 20 days by using phase-contrast microscopy. Scale bar, 100 µm. (**B**) Klf4 protein expression is reduced during TGFβ-induced EMT in NMuMG and Py2T cells. Expression of the epithelial marker E-cadherin and the mesenchymal marker fibronectin as well as Klf4 during a time course of TGFβ-treatment was determined by immunoblotting and GAPDH was used as loading control. (**C**) TGFβ-induced repression of Klf4 expression is dependent on Smad4-mediated TGFβ signaling. Quantitative RT-PCR was performed to monitor the expression levels of Klf4 during TGFβ-mediated EMT in NMuMG cells expressing a control shRNA (shControl) or an shRNA against Smad4 (shSmad4). This experiment was only performed once, hence there is no statistical analysis. **(D–E)** Klf4 mRNA levels were quantified by quantitative RT-PCR after treating NMuMG (D) and Py2T (E) cells with TGFβR inhibitor (TGFβRi), Wnt signaling activator LiCl, EGFR signaling inhibitor (EGFRi) and DMSO (control) in the absence and presence of TGFβ (2 days). Statistical values were calculated using an unpaired/paired, two-tailed t-test and experiments were performed at least three times. * = p≤0.05; *** = p≤0.001.
(TIF)

Figure S2 **Klf4 depletion accelerates TGFβ-induced EMT in Py2T murine breast cancer cells. (A)** Levels of Klf4 protein were determined by immunoblotting in NMuMG and Py2T cells after treatment with Klf4 siRNAs in the absence and presence of TGFβ. The correct band is marked by *Klf4. (**B**) Phase contrast microscopy analysis reveals an acceleration of the morphological changes associated with TGFβ-induced EMT in Klf4-depleted Py2T cells as compared to control siRNA-transfected cells. Size bar, 100 µm. (**C**) Py2T cells were either transfected with control siRNA (siControl) or siRNA against Klf4 (siKlf4). Immunoblotting analysis for the epithelial protein E-cadherin (the correct band is marked with an asterisk) and the mesenchymal proteins N-cadherin and fibronectin reveals an acceleration of TGFβ-induced EMT in Py2T cells after ablation of Klf4 function. Immunoblotting for actin was used as a loading control. **(D)** Quantitative RT-PCR was performed to measure the expression levels of epithelial marker E-Cadherin (Ecad) and mesenchymal markers N-Cadherin (N-Cad) and fibronectin (Fn1) as well as Klf4 during TGFβ-induced EMT in Py2T cells after Klf4 ablation. Statistical values were calculated using an unpaired/paired, two-tailed t-test and experiments were performed at least three times. * = p≤0.05; ** = p≤0.01; *** = p≤0.001.
(TIF)

Figure S3 **Klf4 prevents cell migration and promotes cell survival during TGFβ-induced EMT in Py2T cells. (A, B)** Scratch wound healing assays were performed in NMuMG cells transiently (A) or stably (B) depleted with Klf4 (siKlf4 or shKlf4) and images were captured by phase contrast microscope at 0 hour and at 19 hours after creation of the scratch wounds. Quantification of these experiments are shown in Figure 3A–B. (**C**) siRNA-mediated ablation of Klf4 results in an increase in Py2T cell migration in a trans-well migration assay. siControl and siKlf4 transfected Py2T cells were treated with TGFβ for 15 days, and 20% FBS was used as chemo-attractant. (**D**) Py2T cells transfected with either control siRNA (siControl) or with siRNA against Klf4 (siKlf4) were treated with TGFβ for the days indicated, and the rates of apoptosis were determined by Annexin-V staining and flow cytometry analysis. (**E**) Py2T cells transfected with either control siRNA (siControl) or with siRNA against Klf4 (siKlf4) were treated with TGFβ for the days indicated. Cells were stained with propidium iodide (PI), and the percentages of cells in G0/G1 and S-G2/M phases of the cell cycle were determined by flow cytometry. Statistical values were calculated using an unpaired/paired, two-tailed t-test and experiments were performed at least three times. * = p≤0.05; *** = p≤0.001.
(TIF)

Figure S4 **Klf4 maintains epithelial differentiation and prevents EMT in Py2T cells.** (**A**) Induction of Klf4 transcriptional activity by treatment of Myc-Klf4-ER™-expressing Py2T cells with 4-OHT (+OHT) represses the morphological changes occurring during EMT in control-treated cells (−OHT). Shown are phase contrast images of Myc-Klf4-ER™ expressing Py2T cells treated with TGFβ for 0, 1, 4 and 7 days in the absence or presence of 4-OHT. Size bar, 100 µm. (**B**) Immunoblotting analysis of the expression of the epithelial marker E-cadherin (the correct band is marked with an asterisk) and the mesenchymal markers N-cadherin and fibronectin during TGFβ-induced EMT in Myc-Klf4-ER™-expressing Py2T cells in which Klf4 transcriptional activity has been induced (+OHT) or not (−OHT). Activation of Klf4 in Klf4-ER (+OHT) cells results in the maintenance of the expression of epithelial markers and the failure to express mesenchymal markers. Actin was used as loading control.
(TIF)

Figure S5 **High Klf4 expression correlates with good survival prognosis of breast cancer patients and blocks and metastasis.** (**A, B**) Kaplan-Meier survival analysis reveals a significant correlation of low Klf4 expression levels and poor overall disease-free survival in lymph node-negative (LN−; panel A) and estrogen receptor-negative (ER−; panel B) breast cancer patients (Uppsala database). (**C**) Nuclear localization of Myc-Klf4-ER™ was assessed by immunofluorescence analysis with anti-Myc antibody in non-induced (−OHT) and induced (+OHT) Myc-Klf4-ER™-expressing Py2T cells. Size bar, 50 µm. **(D)** Immunofluorescence analysis was performed to assess the expression levels of the epithelial marker E-cadherin and the mesenchymal markers N-cadherin and fibronectin in tumors derived from normal and Tamoxifen-fed mice after Klf4-ER cells injection in the fat pad of nude mice. (**E**) Myc-Klf4-ER™ and firefly luciferase-expressing Py2T cells were orthotopically transplanted into the mammary fat pad of immune-compromised Balb/c nude mice. Mice were treated with normal food or with food containing Tamoxifen to induce Klf4 transcriptional activity. The percentages of proliferating tumor cells were quantified by immunostaining of histological tumor sections with antibody against phosphorylated histone 3 (pH3). Activation of Klf4 by Tamoxifen had only a moderate effect on tumor cell proliferation. (**F**) Metastatic spread was determined in the tumor-transplanted mice described in (E) by measuring luciferase activity in extracts of lungs and livers of the transplanted mice. Activation of Klf4 by Tamoxifen represses metastatic spread of the transplanted tumor cells. Statistical values were calculated using an unpaired/paired, two-tailed t-test. * = p≤0.05; ** = p≤0.01.
(TIF)

Figure S6 **Klf4 directly binds the promoters of key EMT genes.** (**A–D**) Shown are Venn-diagrams of comparisons between ChIP-seq data after performing Klf4 ChIP (ChIP-Seq), gene expression profiling data from Klf4 knockdown (KD) cells in the absence and presence of TGFβ (2 days), and gene expression profiling data during TGFβ-induced EMT (days 0, 1, 4, 7 and 10). The gene lists originating from a comparison between control and Klf4 knockdown cells, both in the presence and absence of TGFβ, were merged into lists of genes up-regulated (Up) and down-regulated (Dn), respectively, by the loss of Klf4 function. Panels (A) and (B) comprise the genes which were up-regulated during EMT and upon depletion of Klf4 function. Panels (C) and (D) comprise the genes down-regulated during EMT and Klf4 depletion. All experiments were carried out in NMuMG cells. (**E**) Chromatin immunoprecipitation with antibody against Klf4 followed by quantitative PCR (ChIP-qPCR) were performed to demonstrate the occupancy of Klf4 at the promoters of the N-cadherin (Cdh2), vimentin (Vim) and β-catenin (Ctnnb1) genes in Py2T cells. The qPCR data were normalized to ChIP-qPCR of an intergenic region supposed to be free of transcription factor binding. Statistical values were calculated using an unpaired/paired, two-tailed t-test and experiments were performed at least three times. * = p≤0.05.
(TIF)

Figure S7 **Klf4 directly represses Mapk8 (Jnk1) gene expression in Py2T and NMuMG cells and co-depletion of Klf4 and Jnk1 prevents EMT.** (**A**) Ablation of Klf4 function induces Jnk1 expression. Immunoblotting analysis of the expression of Jnk1 during TGFβ-induced EMT in Py2T cells transfected with control siRNA (siControl) or with siRNA against Klf4 (siKlf4). Immunoblotting for actin was used as loading control. (**B, C**) Activation of Klf4 transcriptional activity represses Jnk1 expression. Immunoblotting analysis of Jnk1 expression upon activation of Klf4 by 4-OHT (+OHT) in control NMuMG cells or in Myc-Klf4-ER™-expressing NMuMG cells (B) and in Myc-Klf4-ER™-expressing Py2T (C) cells after treatment with TGFβ for the days indicated. Immunoblotting for actin was used as loading control. (**D**) Klf4 directly binds the Mapk8 (Jnk1) gene promoter in Py2T cells as determined by ChIP using an antibody against Klf4 followed by qPCR with primers specific for the promoter region of the Mapk8 (Jnk1) gene. The qPCR data were normalized to ChIP-qPCR of an intergenic region. (**E**) Quantitative RT-PCR to assess the knockdown efficiency of siRNA against Jnk1 in NMuMG cells transfected with siControl or siKlf4 in the absence and in the presence of TGFβ for 2 days. (**F**) Immunofluorescence microscopy analysis of changes in the localization and expression levels of EMT markers upon depletion of both Klf4 and Jnk1. NMuMG cells transfected with either control siRNA (siControl) or with a combination of siRNAs against Klf4 and Jnk1 (siKlf4+siJnk1) were left untreated or treated with TGFβ for 2 days and stained with antibodies against the epithelial markers E-cadherin and ZO-1, against the mesenchymal markers N-cadherin and fibronectin, against paxillin to detect focal adhesion plaques, and with phalloidin to visualize the actin cytoskeleton. Size bar, 50 µm. Statistical values were calculated using an unpaired/paired, two-tailed t-test and experiments were performed at least three times. ** = p≤0.01; *** = p≤0.001.
(TIF)

Figure S8 **Snail1 and Twist1 are regulated by Klf4 during EMT.** (**A**, **B**) Snail1 (A) and Twist1 (B) mRNA levels were quantified by quantitative RT-PCR after transient depletion of Klf4 in NMuMG cells. Cells were either not treated (day 0) or treated with TGFβ for 2 days (day 2). Shown are the fold-changes as compared to day 0 (no TGFβ). Statistical values were calculated using an unpaired/paired, two-tailed t-test and experiments were performed at least three times. * = p≤0.05.
(TIF)

Figure S9 **Working model of Klf4 function during EMT.** Klf4 maintains the epithelial differentiation status by activating the expression of epithelial genes, such as E-cadherin, and by repressing the expression of mesenchymal genes, such as Mapk8 (Jnk1), N-cadherin, vimentin, and β-catenin. Upon TGFβ-induced Smad4-dependent repression of Klf4 expression, epithelial genes are no more activated by Klf4 and mesenchymal genes are de-repressed, together leading to EMT.
(TIF)

Table S1 **The table represents a list of genes that are directly bound by Klf4 and that are either up-regulated or down-regulated in their expression by the loss of Klf4 function and during the various stages of TGFβ-induced EMT in NMuMG cell.**
(JPG)

Table S2 **Gene-Ontology analysis reveals that crucial EMT relevant pathways are under control of Klf4.** GeneGo software was used to categorize the genes into functional groups. Gene ontology has been performed on direct KLf4 target genes that are transcriptionally up or down-regulated during EMT.
(XLSX)
