# Correction: Klf4 Is a Transcriptional Regulator of Genes Critical for EMT, Including Jnk1 (*Mapk8*)

**DOI:** 10.1371/annotation/121b04a1-0cbb-4e24-8a63-fc9cdd31ec76

**Published:** 2013-10-25

**Authors:** Neha Tiwari, Nathalie Meyer-Schaller, Phil Arnold, Helena Antoniadis, Mikhail Pachkov, Erik van Nimwegen, Gerhard Christofori

There was an error in the last sentence of the "Microarray Processing and Data Analysis" section of the Materials and Methods. The last sentence should read: "The microarray data has been deposited on ArrayExpress platform under the accession number GSE49451." 

