# Supplementary figures and images for: Klf4 Is a Transcriptional Regulator of Genes Critical for EMT, Including Jnk1 (Mapk8)
Source: PLoS One. 2013 Feb 25;8(2):e57329. doi: 10.1371/journal.pone.0057329 (PMC3581489; doi:10.1371/journal.pone.0057329)

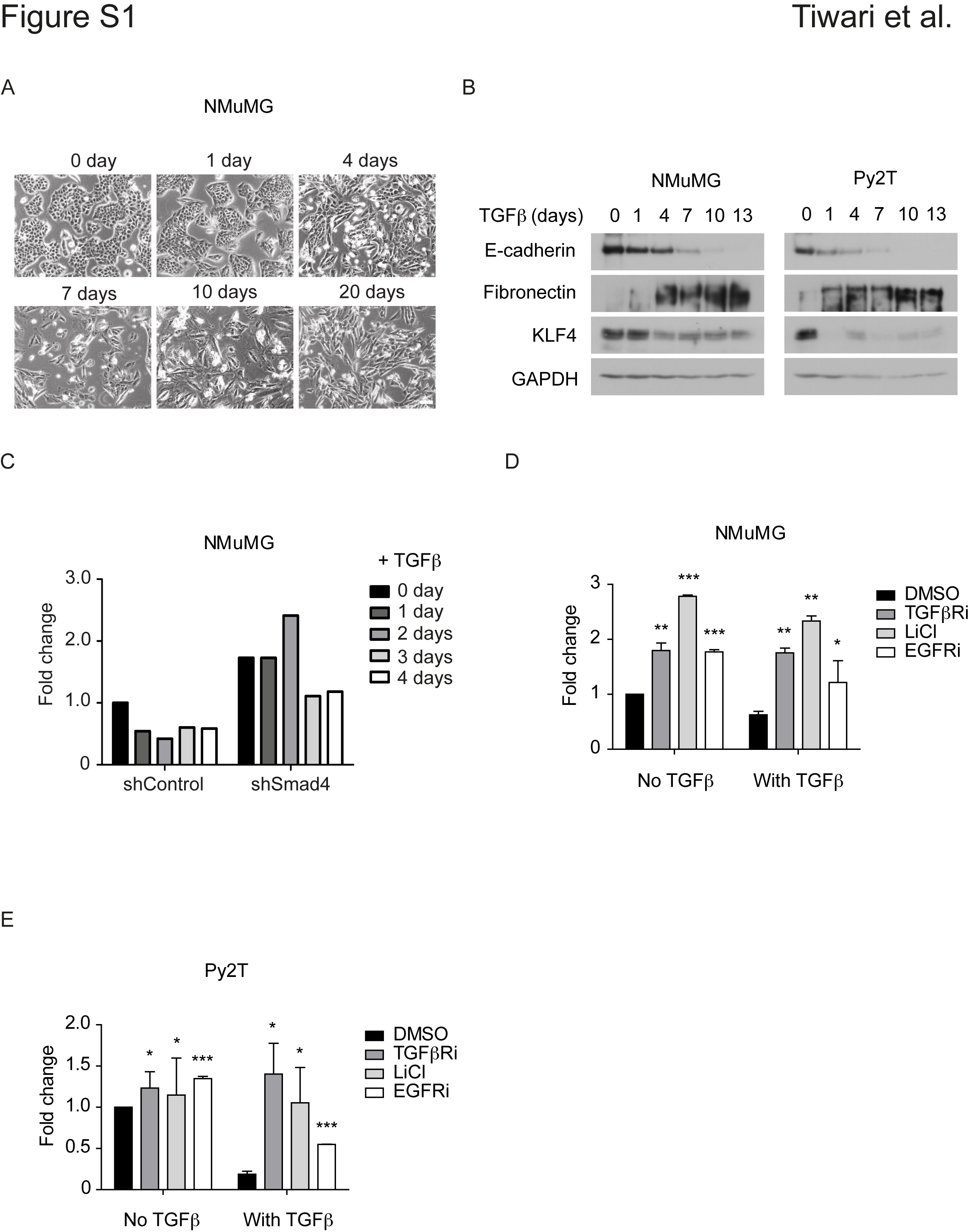

Supplement: Figure S1 — Klf4 expression is repressed by canonical TGFβ signaling during TGFβ-induced EMT in NMuMG cells. (A) Morphological changes observed in NMuMG cells treated with TGFβ for 0, 1, 4, 7, 10 and 20 days by using phase-contrast microscopy. Scale bar, 100 µm. (B) Klf4 protein expression is reduced during TGFβ-induced EMT in NMuMG and Py2T cells. Expression of the epithelial marker E-cadherin and the mesenchymal marker fibronectin as well as Klf4 during a time course of TGFβ-treatment was determined by immunoblotting and GAPDH was used as loading control. (C) TGFβ-induced repression of Klf4 expression is dependent on Smad4-mediated TGFβ signaling. Quantitative RT-PCR was performed to monitor the expression levels of Klf4 during TGFβ-mediated EMT in NMuMG cells expressing a control shRNA (shControl) or an shRNA against Smad4 (shSmad4). This experiment was only performed once, hence there is no statistical analysis. (D–E) Klf4 mRNA levels were quantified by quantitative RT-PCR after treating NMuMG (D) and Py2T (E) cells with TGFβR inhibitor (TGFβRi), Wnt signaling activator LiCl, EGFR signaling inhibitor (EGFRi) and DMSO (control) in the absence and presence of TGFβ (2 days). Statistical values were calculated using an unpaired/paired, two-tailed t-test and experiments were performed at least three times. * = p≤0.05; *** = p≤0.001. (TIF) [file pone.0057329.s001.tif]

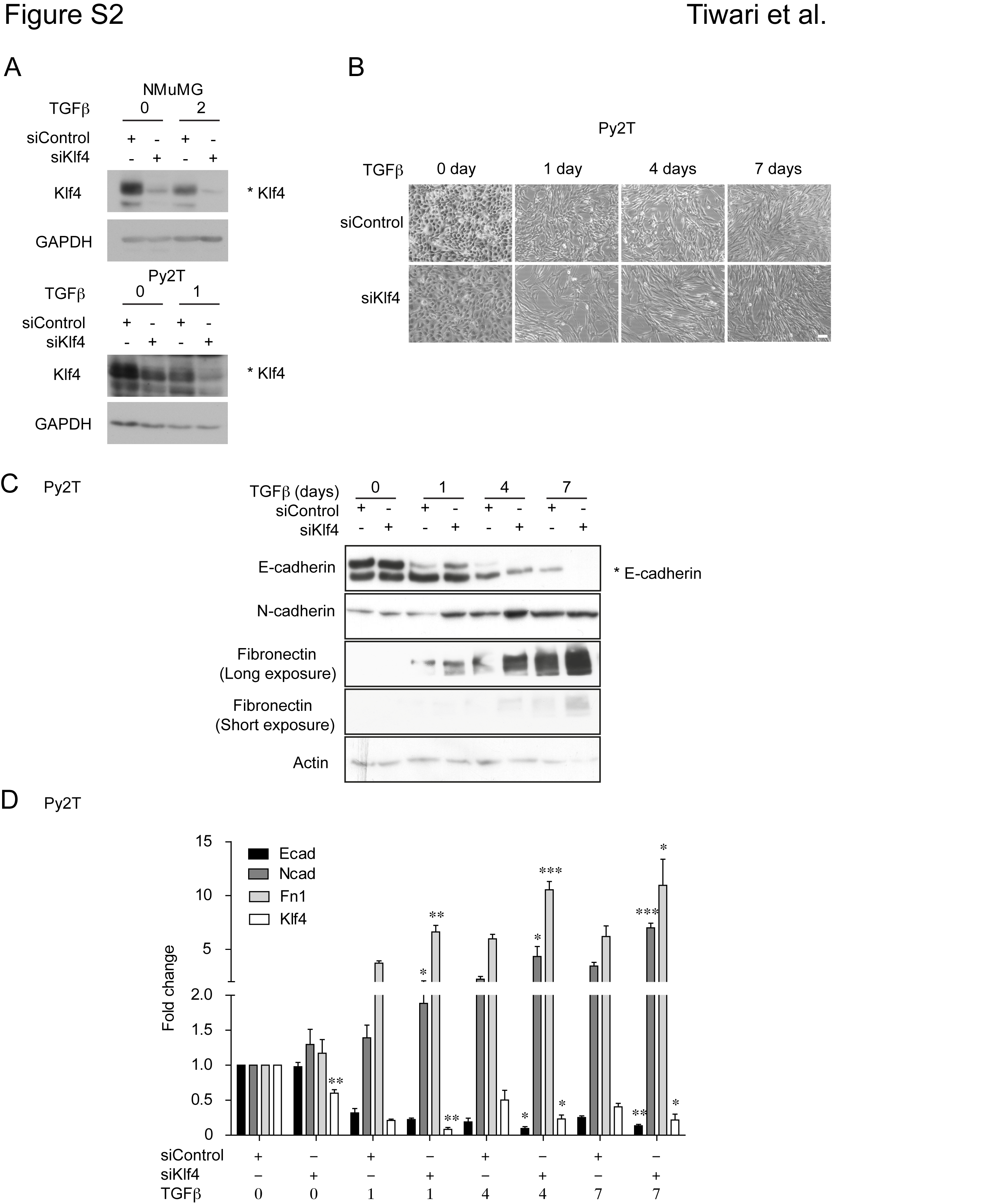

Supplement: Figure S2 — Klf4 depletion accelerates TGFβ-induced EMT in Py2T murine breast cancer cells. (A) Levels of Klf4 protein were determined by immunoblotting in NMuMG and Py2T cells after treatment with Klf4 siRNAs in the absence and presence of TGFβ. The correct band is marked by *Klf4. (B) Phase contrast microscopy analysis reveals an acceleration of the morphological changes associated with TGFβ-induced EMT in Klf4-depleted Py2T cells as compared to control siRNA-transfected cells. Size bar, 100 µm. (C) Py2T cells were either transfected with control siRNA (siControl) or siRNA against Klf4 (siKlf4). Immunoblotting analysis for the epithelial protein E-cadherin (the correct band is marked with an asterisk) and the mesenchymal proteins N-cadherin and fibronectin reveals an acceleration of TGFβ-induced EMT in Py2T cells after ablation of Klf4 function. Immunoblotting for actin was used as a loading control. (D) Quantitative RT-PCR was performed to measure the expression levels of epithelial marker E-Cadherin (Ecad) and mesenchymal markers N-Cadherin (N-Cad) and fibronectin (Fn1) as well as Klf4 during TGFβ-induced EMT in Py2T cells after Klf4 ablation. Statistical values were calculated using an unpaired/paired, two-tailed t-test and experiments were performed at least three times. * = p≤0.05; ** = p≤0.01; *** = p≤0.001. (TIF) [file pone.0057329.s002.tif]

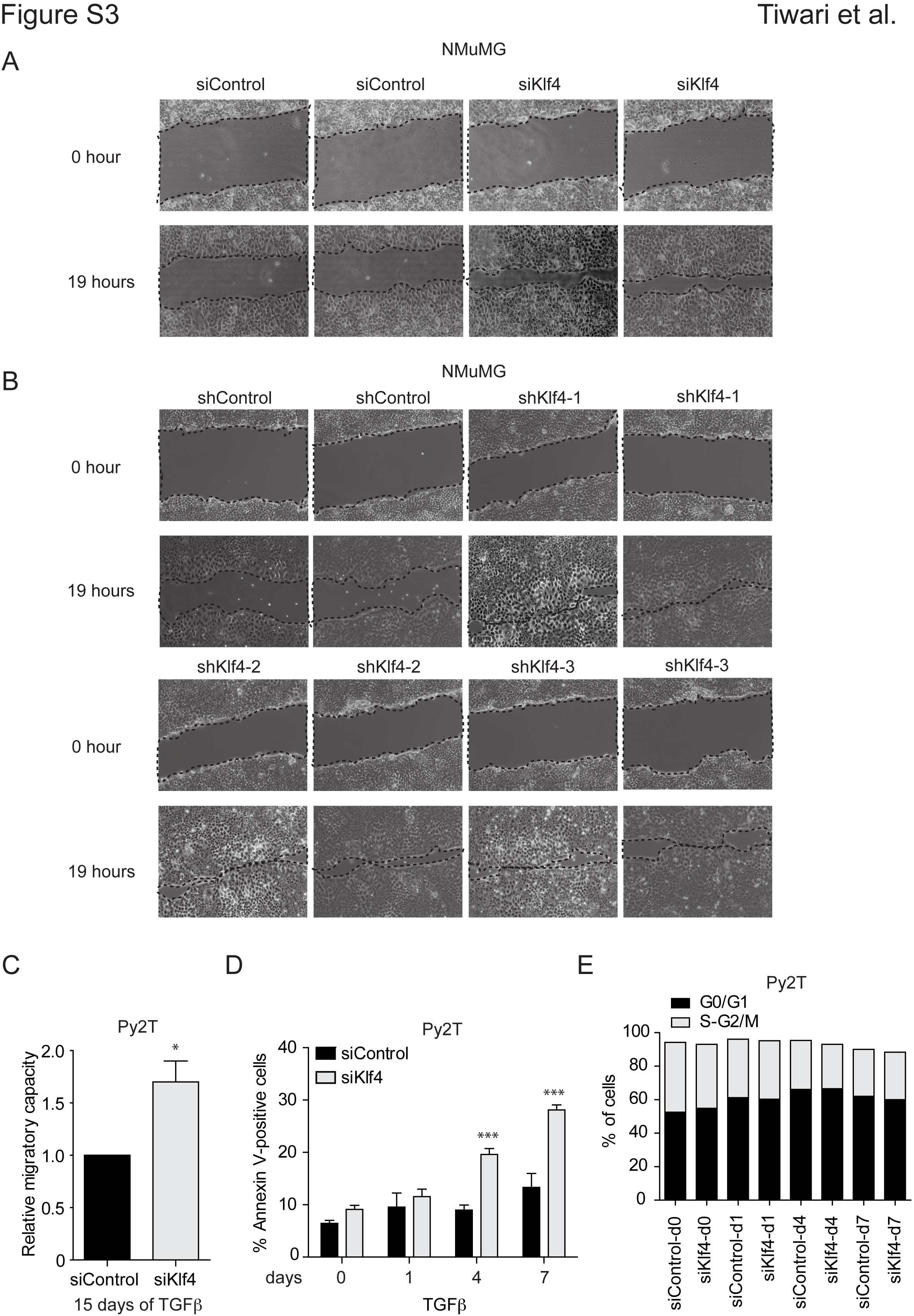

Supplement: Figure S3 — Klf4 prevents cell migration and promotes cell survival during TGFβ-induced EMT in Py2T cells. (A, B) Scratch wound healing assays were performed in NMuMG cells transiently (A) or stably (B) depleted with Klf4 (siKlf4 or shKlf4) and images were captured by phase contrast microscope at 0 hour and at 19 hours after creation of the scratch wounds. Quantification of these experiments are shown in Figure 3A–B. (C) siRNA-mediated ablation of Klf4 results in an increase in Py2T cell migration in a trans-well migration assay. siControl and siKlf4 transfected Py2T cells were treated with TGFβ for 15 days, and 20% FBS was used as chemo-attractant. (D) Py2T cells transfected with either control siRNA (siControl) or with siRNA against Klf4 (siKlf4) were treated with TGFβ for the days indicated, and the rates of apoptosis were determined by Annexin-V staining and flow cytometry analysis. (E) Py2T cells transfected with either control siRNA (siControl) or with siRNA against Klf4 (siKlf4) were treated with TGFβ for the days indicated. Cells were stained with propidium iodide (PI), and the percentages of cells in G0/G1 and S-G2/M phases of the cell cycle were determined by flow cytometry. Statistical values were calculated using an unpaired/paired, two-tailed t-test and experiments were performed at least three times. * = p≤0.05; *** = p≤0.001. (TIF) [file pone.0057329.s003.tif]

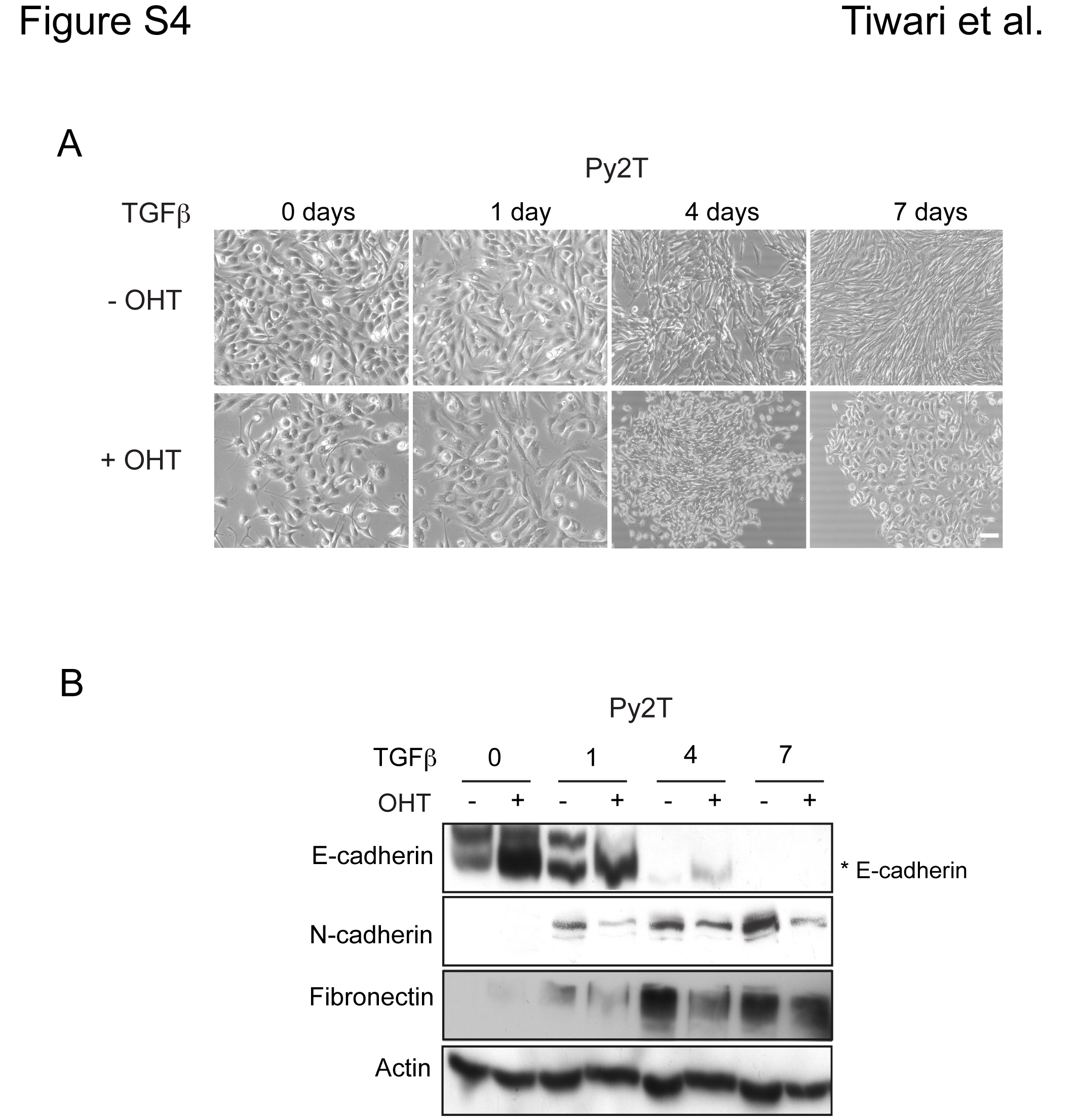

Supplement: Figure S4 — Klf4 maintains epithelial differentiation and prevents EMT in Py2T cells. (A) Induction of Klf4 transcriptional activity by treatment of Myc-Klf4-ER™-expressing Py2T cells with 4-OHT (+OHT) represses the morphological changes occurring during EMT in control-treated cells (−OHT). Shown are phase contrast images of Myc-Klf4-ER™ expressing Py2T cells treated with TGFβ for 0, 1, 4 and 7 days in the absence or presence of 4-OHT. Size bar, 100 µm. (B) Immunoblotting analysis of the expression of the epithelial marker E-cadherin (the correct band is marked with an asterisk) and the mesenchymal markers N-cadherin and fibronectin during TGFβ-induced EMT in Myc-Klf4-ER™-expressing Py2T cells in which Klf4 transcriptional activity has been induced (+OHT) or not (−OHT). Activation of Klf4 in Klf4-ER (+OHT) cells results in the maintenance of the expression of epithelial markers and the failure to express mesenchymal markers. Actin was used as loading control. (TIF) [file pone.0057329.s004.tif]

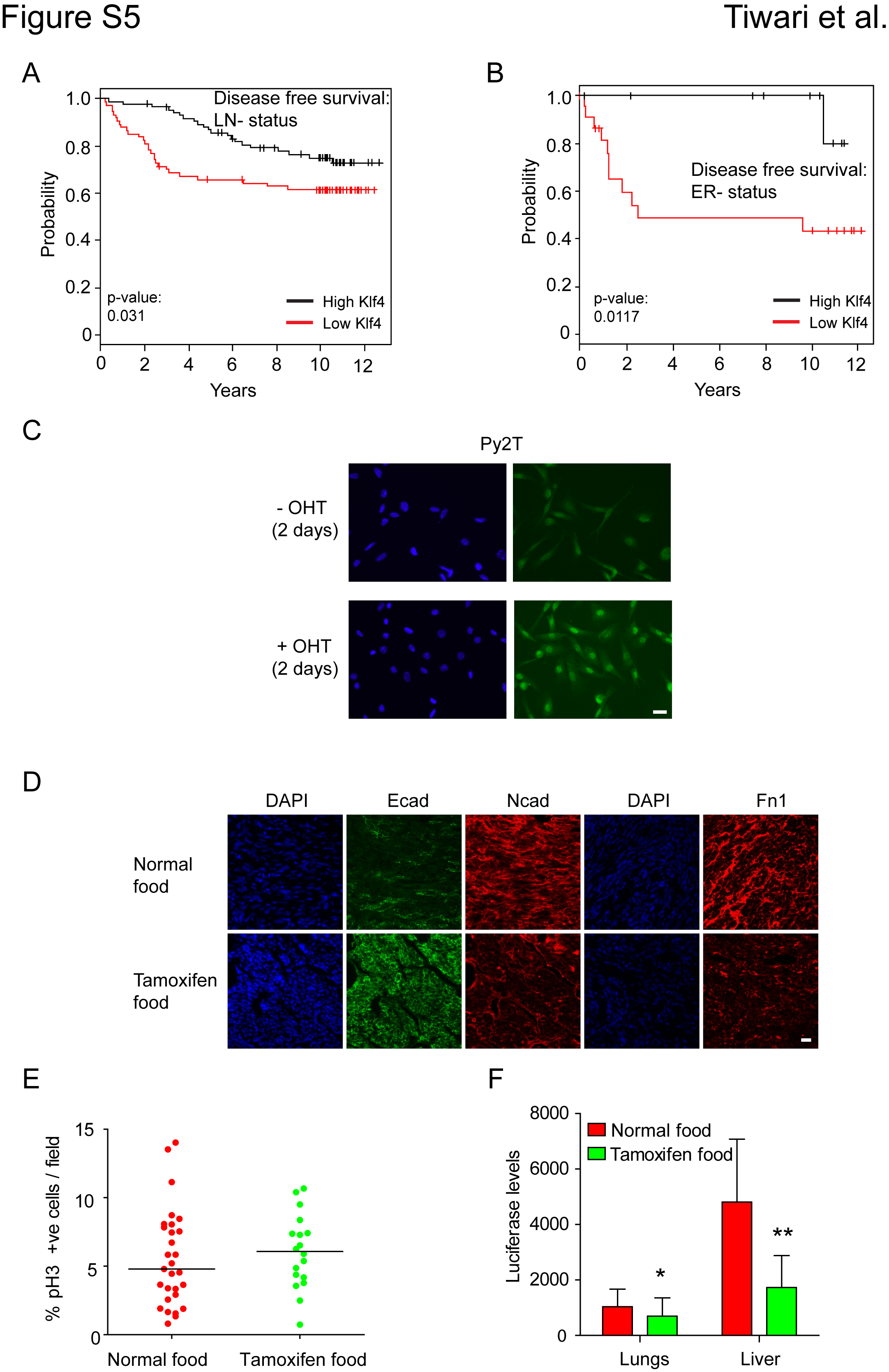

Supplement: Figure S5 — High Klf4 expression correlates with good survival prognosis of breast cancer patients and blocks and metastasis. (A, B) Kaplan-Meier survival analysis reveals a significant correlation of low Klf4 expression levels and poor overall disease-free survival in lymph node-negative (LN−; panel A) and estrogen receptor-negative (ER−; panel B) breast cancer patients (Uppsala database). (C) Nuclear localization of Myc-Klf4-ER™ was assessed by immunofluorescence analysis with anti-Myc antibody in non-induced (−OHT) and induced (+OHT) Myc-Klf4-ER™-expressing Py2T cells. Size bar, 50 µm. (D) Immunofluorescence analysis was performed to assess the expression levels of the epithelial marker E-cadherin and the mesenchymal markers N-cadherin and fibronectin in tumors derived from normal and Tamoxifen-fed mice after Klf4-ER cells injection in the fat pad of nude mice. (E) Myc-Klf4-ER™ and firefly luciferase-expressing Py2T cells were orthotopically transplanted into the mammary fat pad of immune-compromised Balb/c nude mice. Mice were treated with normal food or with food containing Tamoxifen to induce Klf4 transcriptional activity. The percentages of proliferating tumor cells were quantified by immunostaining of histological tumor sections with antibody against phosphorylated histone 3 (pH3). Activation of Klf4 by Tamoxifen had only a moderate effect on tumor cell proliferation. (F) Metastatic spread was determined in the tumor-transplanted mice described in (E) by measuring luciferase activity in extracts of lungs and livers of the transplanted mice. Activation of Klf4 by Tamoxifen represses metastatic spread of the transplanted tumor cells. Statistical values were calculated using an unpaired/paired, two-tailed t-test. * = p≤0.05; ** = p≤0.01. (TIF) [file pone.0057329.s005.tif]

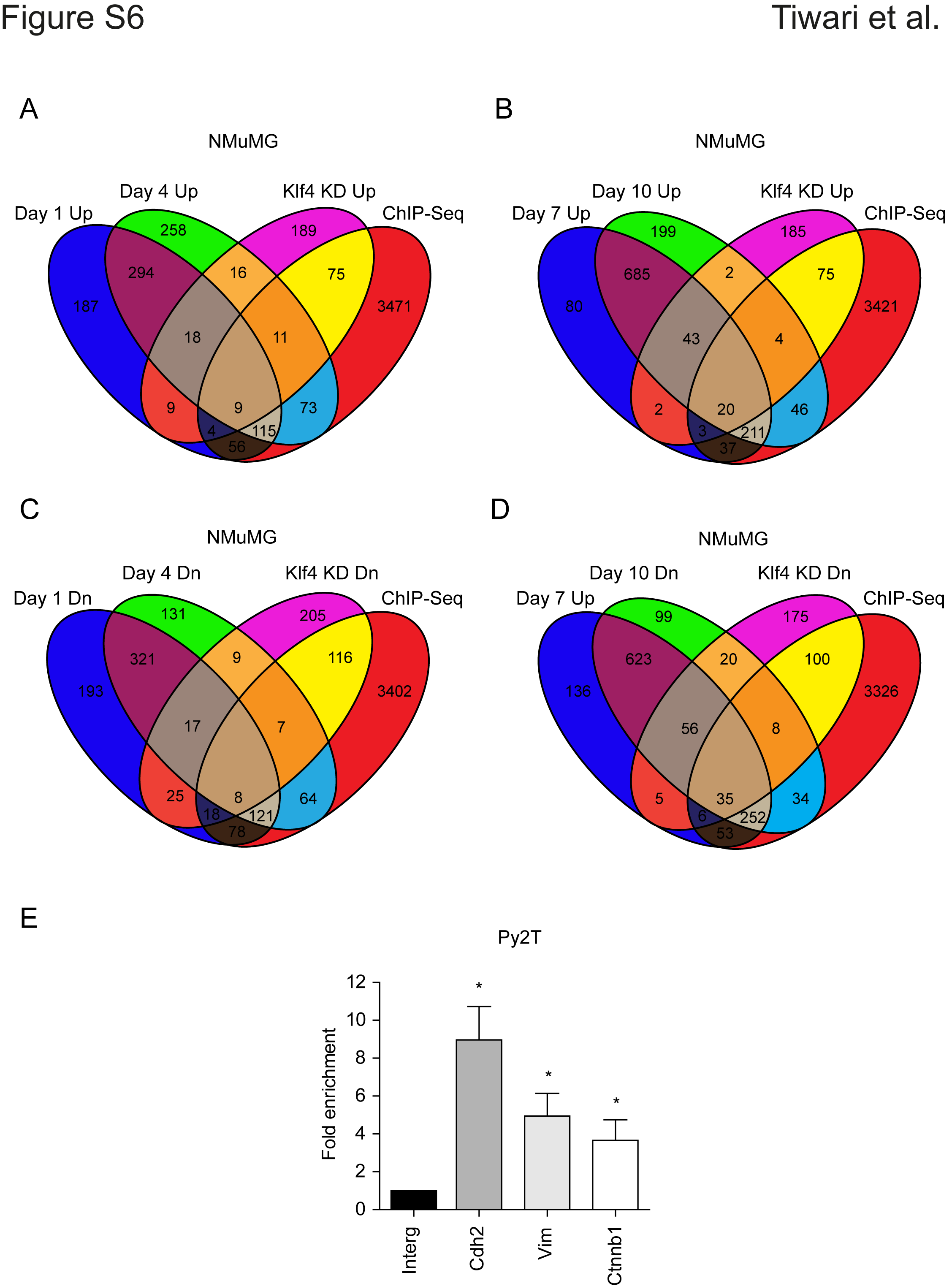

Supplement: Figure S6 — Klf4 directly binds the promoters of key EMT genes. (A–D) Shown are Venn-diagrams of comparisons between ChIP-seq data after performing Klf4 ChIP (ChIP-Seq), gene expression profiling data from Klf4 knockdown (KD) cells in the absence and presence of TGFβ (2 days), and gene expression profiling data during TGFβ-induced EMT (days 0, 1, 4, 7 and 10). The gene lists originating from a comparison between control and Klf4 knockdown cells, both in the presence and absence of TGFβ, were merged into lists of genes up-regulated (Up) and down-regulated (Dn), respectively, by the loss of Klf4 function. Panels (A) and (B) comprise the genes which were up-regulated during EMT and upon depletion of Klf4 function. Panels (C) and (D) comprise the genes down-regulated during EMT and Klf4 depletion. All experiments were carried out in NMuMG cells. (E) Chromatin immunoprecipitation with antibody against Klf4 followed by quantitative PCR (ChIP-qPCR) were performed to demonstrate the occupancy of Klf4 at the promoters of the N-cadherin (Cdh2), vimentin (Vim) and β-catenin (Ctnnb1) genes in Py2T cells. The qPCR data were normalized to ChIP-qPCR of an intergenic region supposed to be free of transcription factor binding. Statistical values were calculated using an unpaired/paired, two-tailed t-test and experiments were performed at least three times. * = p≤0.05. (TIF) [file pone.0057329.s006.tif]

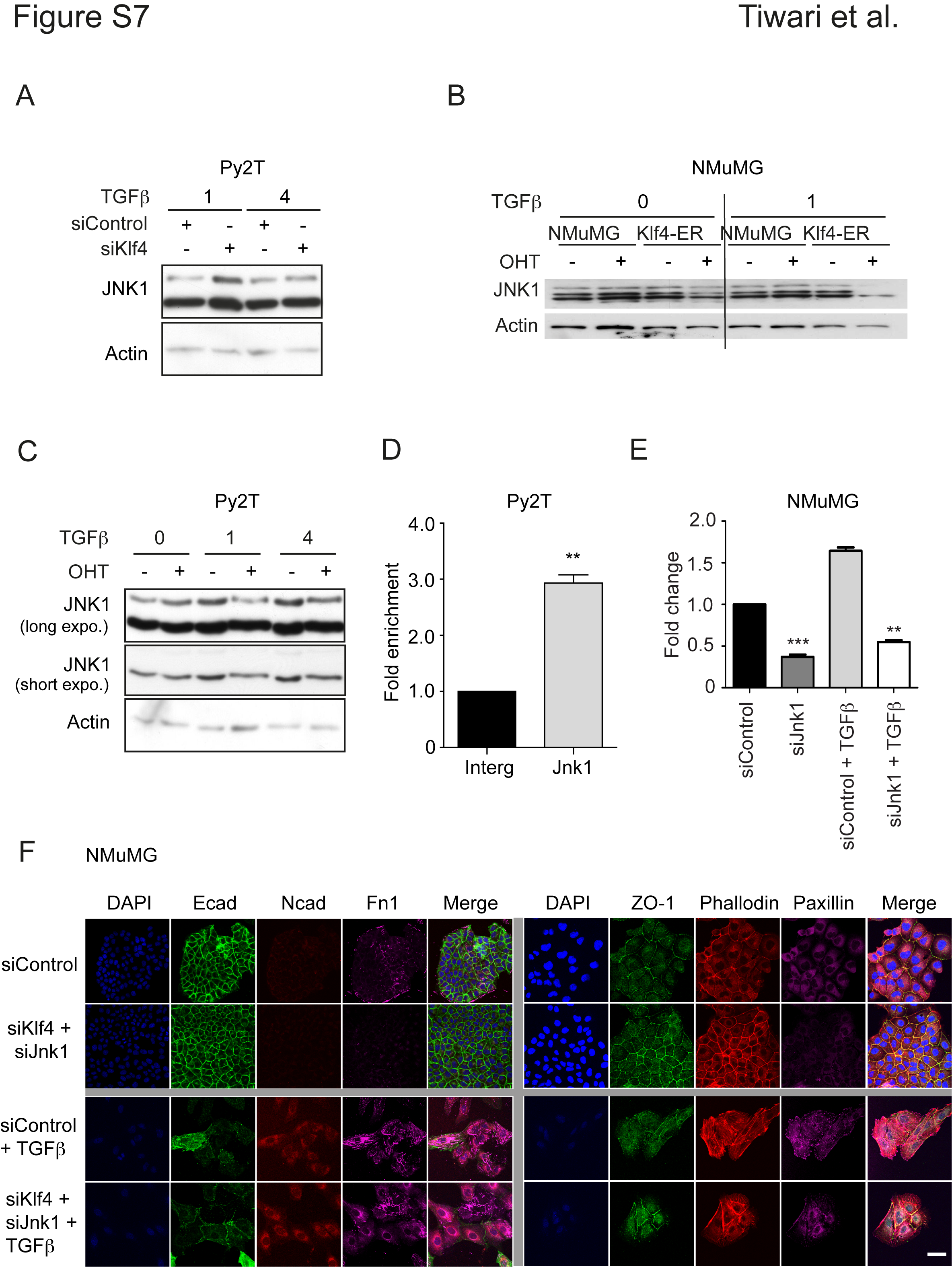

Supplement: Figure S7 — Klf4 directly represses Mapk8 (Jnk1) gene expression in Py2T and NMuMG cells and co-depletion of Klf4 and Jnk1 prevents EMT. (A) Ablation of Klf4 function induces Jnk1 expression. Immunoblotting analysis of the expression of Jnk1 during TGFβ-induced EMT in Py2T cells transfected with control siRNA (siControl) or with siRNA against Klf4 (siKlf4). Immunoblotting for actin was used as loading control. (B, C) Activation of Klf4 transcriptional activity represses Jnk1 expression. Immunoblotting analysis of Jnk1 expression upon activation of Klf4 by 4-OHT (+OHT) in control NMuMG cells or in Myc-Klf4-ER™-expressing NMuMG cells (B) and in Myc-Klf4-ER™-expressing Py2T (C) cells after treatment with TGFβ for the days indicated. Immunoblotting for actin was used as loading control. (D) Klf4 directly binds the Mapk8 (Jnk1) gene promoter in Py2T cells as determined by ChIP using an antibody against Klf4 followed by qPCR with primers specific for the promoter region of the Mapk8 (Jnk1) gene. The qPCR data were normalized to ChIP-qPCR of an intergenic region. (E) Quantitative RT-PCR to assess the knockdown efficiency of siRNA against Jnk1 in NMuMG cells transfected with siControl or siKlf4 in the absence and in the presence of TGFβ for 2 days. (F) Immunofluorescence microscopy analysis of changes in the localization and expression levels of EMT markers upon depletion of both Klf4 and Jnk1. NMuMG cells transfected with either control siRNA (siControl) or with a combination of siRNAs against Klf4 and Jnk1 (siKlf4+siJnk1) were left untreated or treated with TGFβ for 2 days and stained with antibodies against the epithelial markers E-cadherin and ZO-1, against the mesenchymal markers N-cadherin and fibronectin, against paxillin to detect focal adhesion plaques, and with phalloidin to visualize the actin cytoskeleton. Size bar, 50 µm. Statistical values were calculated using an unpaired/paired, two-tailed t-test and experiments were performed at least three times. ** = p≤0.01; *** = p≤ [file pone.0057329.s007.tif]

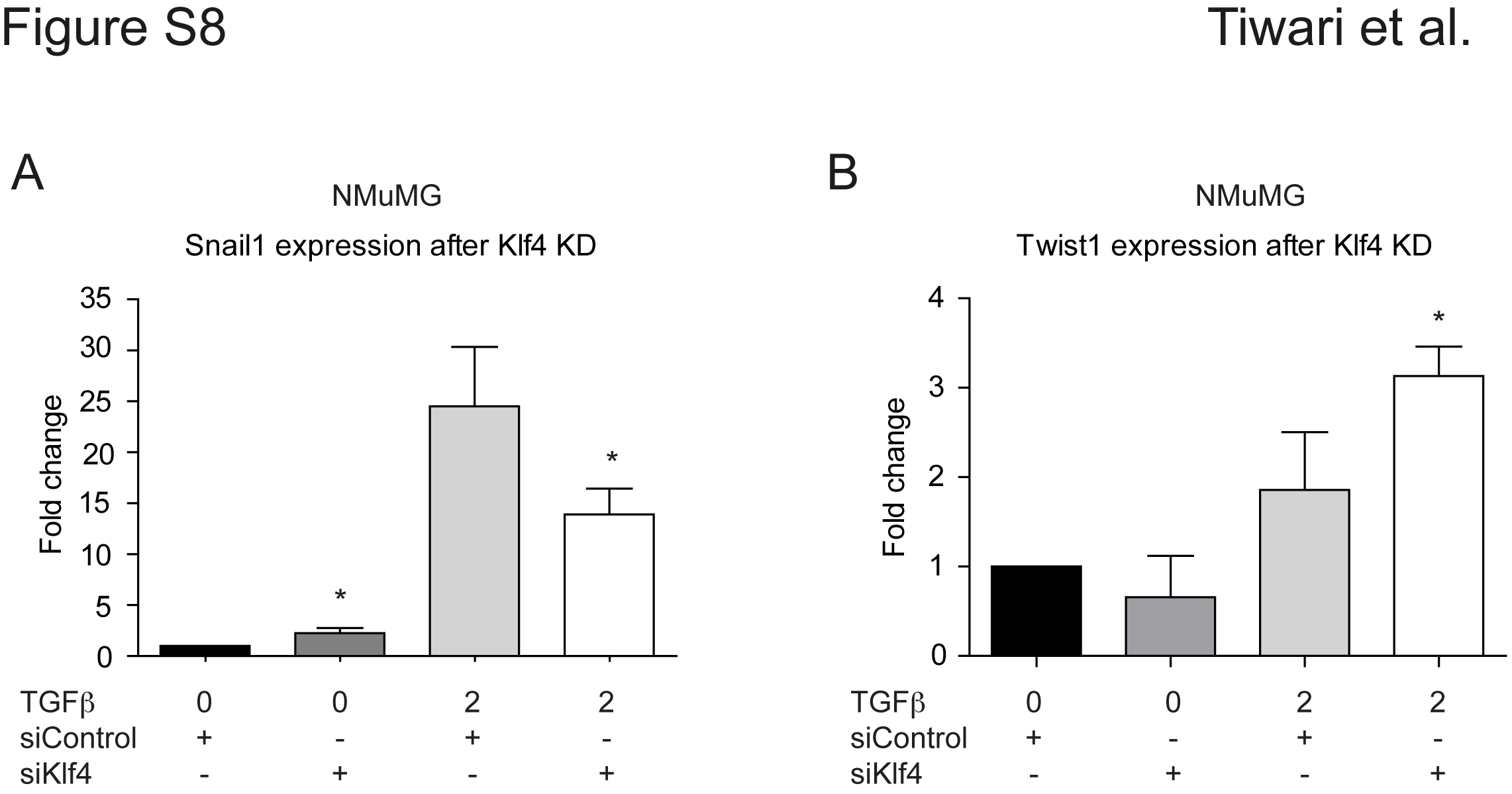

Supplement: Figure S8 — Snail1 and Twist1 are regulated by Klf4 during EMT. (A, B) Snail1 (A) and Twist1 (B) mRNA levels were quantified by quantitative RT-PCR after transient depletion of Klf4 in NMuMG cells. Cells were either not treated (day 0) or treated with TGFβ for 2 days (day 2). Shown are the fold-changes as compared to day 0 (no TGFβ). Statistical values were calculated using an unpaired/paired, two-tailed t-test and experiments were performed at least three times. * = p≤0.05. (TIF) [file pone.0057329.s008.tif]

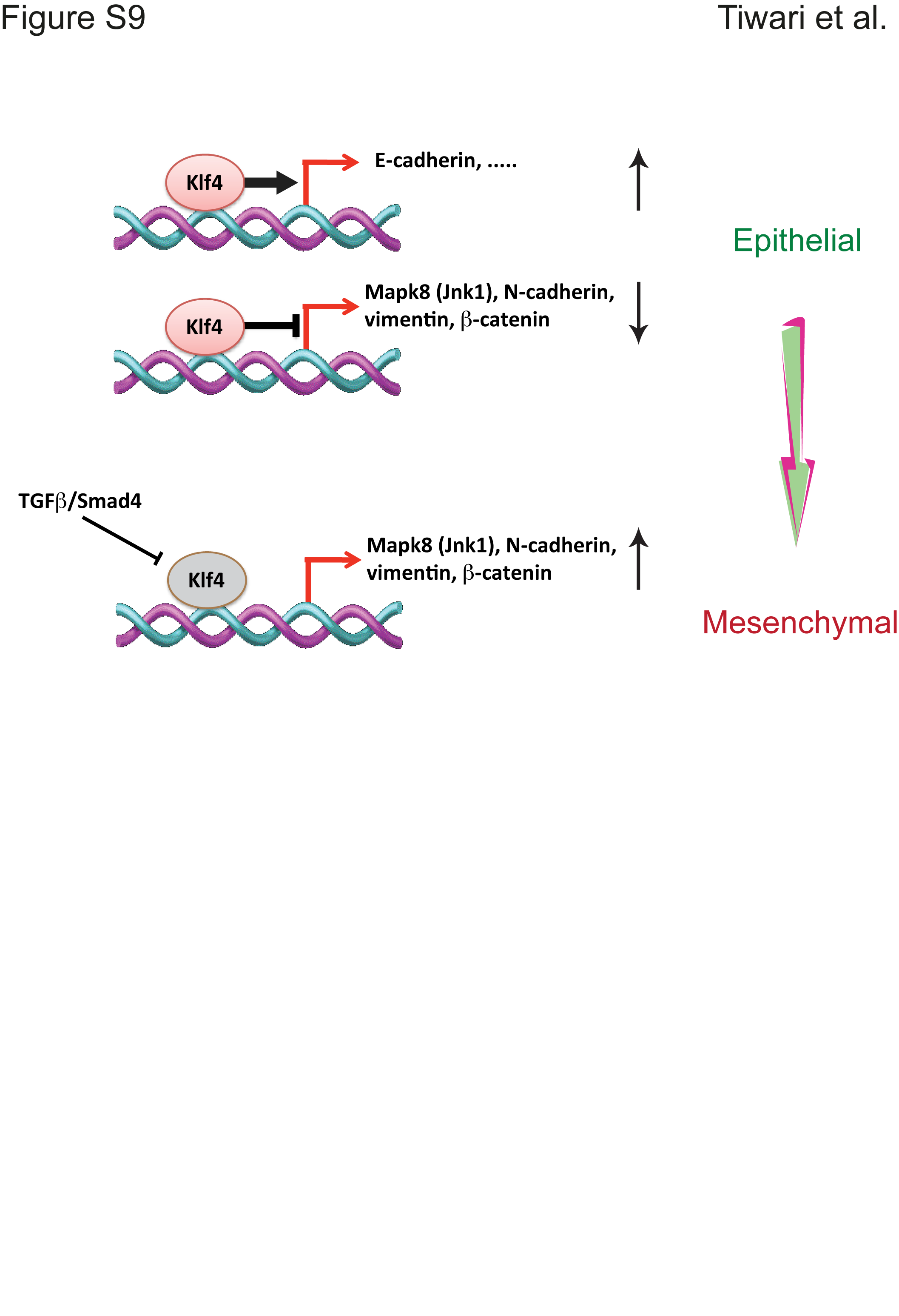

Supplement: Figure S9 — Working model of Klf4 function during EMT. Klf4 maintains the epithelial differentiation status by activating the expression of epithelial genes, such as E-cadherin, and by repressing the expression of mesenchymal genes, such as Mapk8 (Jnk1), N-cadherin, vimentin, and β-catenin. Upon TGFβ-induced Smad4-dependent repression of Klf4 expression, epithelial genes are no more activated by Klf4 and mesenchymal genes are de-repressed, together leading to EMT. (TIF) [file pone.0057329.s009.tif]

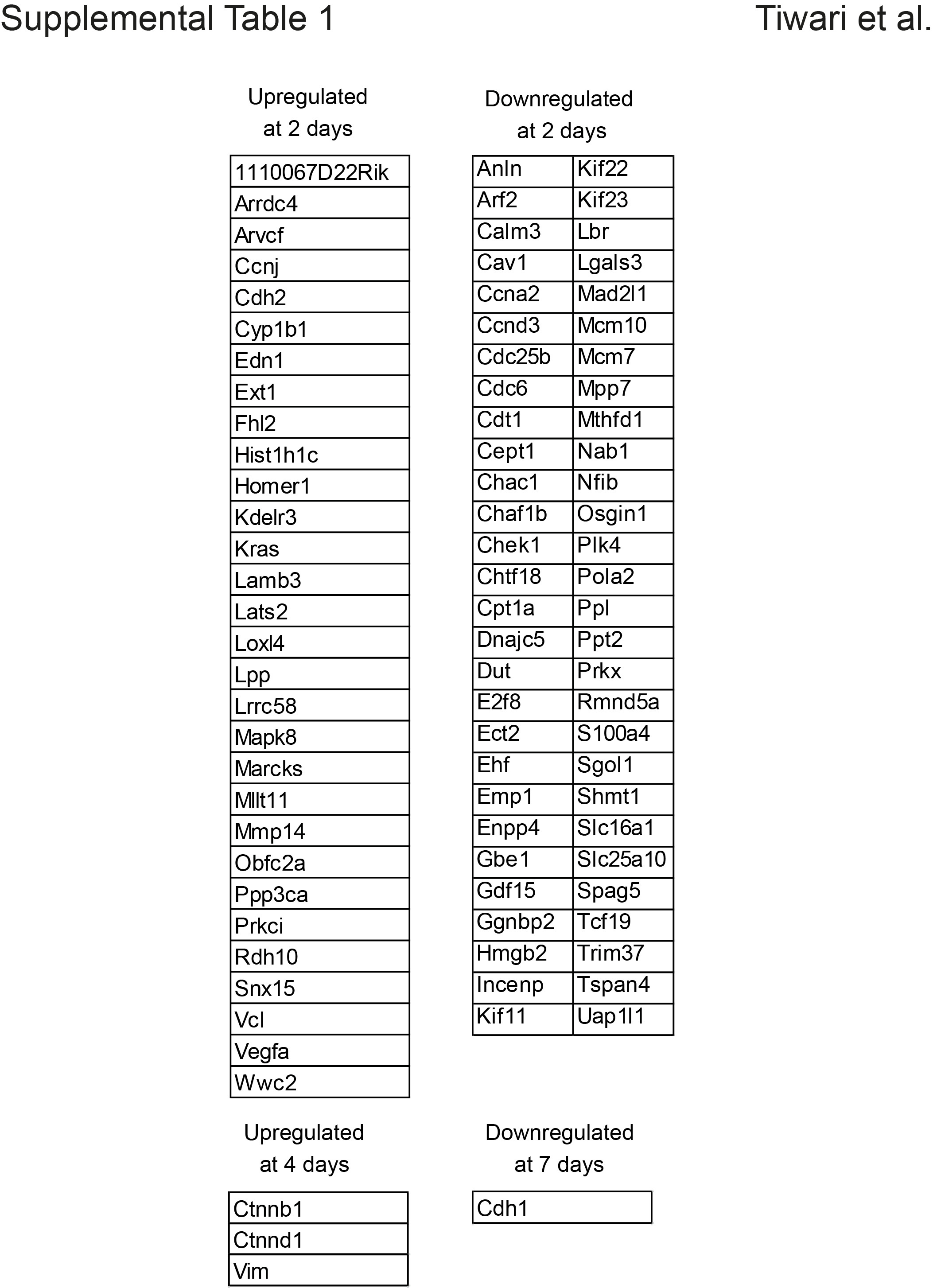

Supplement: Table S1 — The table represents a list of genes that are directly bound by Klf4 and that are either up-regulated or down-regulated in their expression by the loss of Klf4 function and during the various stages of TGFβ-induced EMT in NMuMG cell. (JPG) [file pone.0057329.s010.jpg]
